# Supplementary material for: What is multidisciplinary cancer care like in practice? a protocol for a mixed-method study to characterise ambulatory oncology services in the Australian public sector
Source: BMJ Open. 2019 Oct 10;9(10):e031179. doi: 10.1136/bmjopen-2019-031179 (PMC6797275; doi:10.1136/bmjopen-2019-031179)
Supplement: Author's manuscript [file bmjopen-2019-031179.draft_revisions.pdf]

BMJ Open is committed to open peer review. As part of this commitment we make the peer review history of every article we publish publicly available.

When an article is published we post the peer reviewers' comments and the authors' responses online. We also post the versions of the paper that were used during peer review. These are the versions that the peer review comments apply to.

The versions of the paper that follow are the versions that were submitted during the peer review process. They are not the versions of record or the final published versions. They should not be cited or distributed as the published version of this manuscript.

BMJ Open is an open access journal and the full, final, typeset and author-corrected version of record of the manuscript is available on our site with no access controls, subscription charges or pay-per-view fees (http://bmjopen.bmj.com).

If you have any questions on BMJ Open's open peer review process please email info.bmjopen@bmj.com

BMJ Open

What is Multidisciplinary Cancer Care Like in Practice? A protocol for a mixed-method study to characterise ambulatory oncology services in the public sector

Journal:	*BMJ Open*
Manuscript ID	bmjopen-2019-031179
Article Type:	Protocol
Date Submitted by the Author:	20-Apr-2019

Complete List of Authors:	Nic Giolla Easpaig, Bróna; Macquarie University, Australian Institute of Health Innovation<br>Arnolda, Gaston; Macquarie University, Australian Institute of Health Innovation<br>Tran, Yvonne ; Macquarie University, Australian Institute of Health Innovation<br>Bierbaum, Mia; Macquarie University, Australian Institute of Health Innovation<br>Lamprell, Klay; Macquarie University, Australian Institute of Health Innovation<br>Delaney, Geoffrey; Liverpool Cancer Therapy Centre; University of New South Wales South Western Sydney Clinical School<br>Liauw, Winston; Saint George Hospital Saint George Cancer Care Centre; University of New South Wales, St George Hospital Clinical School<br>Chittajallu, Renuka; Macquarie University, Australian Institute of Health Innovation<br>Winata, Teresa; Macquarie University, Australian Institute of Health Innovation; Griffith University,<br>Ward, Robyn; University of Sydney, Faculty of Medicine and Health; University of New South Wales, Prince of Wales Clinical School<br>Currow, David ; Flinders University, College of Medicine and Public Health; University of Technology Sydney, Faculty of Health<br>Olver, Ian; University of South Australia, Division of Health Sciences<br>Karnon, Jonathan; Flinders University, College of Medicine and Public Health<br>Westbrook, Johanna; Macquarie University, Australian Institute of Health Innovation<br>Braithwaite, Jeffrey; Macquarie University, Australian Institute of Health Innovation

Keywords:	cancer, QUALITATIVE RESEARCH, multi-site, ONCOLOGY, outpatient clinic

SCHOLARONE™
Manuscripts

Title: **What is Multidisciplinary Cancer Care Like in Practice? A protocol for a mixed-method study to characterise ambulatory oncology services in the public sector**

Authors:

1. Nic Giolla Easpaig, Bróna (Corresponding author)

   Postal Address: Australian Institute of Health Innovation, Macquarie University Level 6, 75 Talavera Road, Sydney, NSW 2109, Australia

   Email: brona.nicgiollaeaspaig@mq.edu.au

   Phone number: +61 2 9850 2400

2. Arnolda, Gaston

   Australian Institute of Health Innovation, Macquarie University, NSW, Sydney, Australia

3. Tran, Yvonne

   Australian Institute of Health Innovation, Macquarie University, NSW, Sydney, Australia

4. Bierbaum, Mia

   Australian Institute of Health Innovation, Macquarie University, Sydney, NSW, Australia

5. Lamprell, Klay,

   Australian Institute of Health Innovation, Macquarie University, Sydney, NSW, Australia

6. Delaney, Geoffrey, P.

   i. Liverpool Cancer Therapy Centre, Liverpool, NSW, Australia

   ii. South Western Sydney Clinical School, University of New South Wales, Liverpool, NSW, Australia

For peer review only - http://bmjopen.bmj.com/site/about/guidelines.xhtml

7. Liauw, Winston,

   i. St. George Cancer Care Centre, St. George Hospital, Kogarah, NSW, Australia
   ii. St. George Hospital Clinical School, University of New South Wales, Sydney, NSW, Australia

8. Chittajallu, Renuka

   Australian Institute of Health Innovation, Macquarie University, Sydney, NSW, Australia

9. Winata, Teresa

   Australian Institute of Health Innovation, Macquarie University, Sydney, NSW, Australia

10. Ward, Robyn, L.

   i. Faculty of Medicine and Health, The University of Sydney, Sydney, NSW, Australia
   ii. Prince of Wales Clinical School, University of New South Wales, Sydney, NSW, Australia

11. Currow, David

   i. College of Medicine and Public Health, Flinders University, Adelaide, SA, Australia
   ii. Faculty of Health, University of Technology Sydney, Sydney, NSW, Australia

12. Olver, Ian,

   Division of Health Sciences, University of South Australia, Adelaide, SA, Australia

13. Karnon, Jon,

   College of Medicine and Public Health, Flinders University, Adelaide, SA, Australia

14. Westbrook, Johanna

For peer review only - http://bmjopen.bmj.com/site/about/guidelines.xhtml

Australian Institute of Health Innovation, Macquarie University, NSW, Sydney, Australia

15. Braithwaite, Jeffrey

Australian Institute of Health Innovation, Macquarie University, Sydney, NSW, Australia

Word count: 3,992 (excluding title page, abstract, references, figures and tables)

Keywords: qualitative research; multi-site; oncology; cancer; outpatient clinic

For peer review only

ABSTRACT

Introduction

An understanding of the real-world provision of oncology outpatient services can help maintain service quality in the face of escalating demand and tight budgets, by informing the design of interventions that improve the effectiveness or efficiency of provision. The aims of this study are threefold. First, to develop an understanding of cancer services in outpatient clinics by characterising the organisation and practice of multidisciplinary care (MDC). Second, to explore the key areas of: a) clinical decision-making; and b) engagement with patients' supportive needs. Third, to identify barriers to, and facilitators of, the delivery of quality care in these settings.

Methods and analysis

A suite of mixed-methods studies will be implemented at six hospitals providing cancer outpatient clinics (OPCs), with a staged roll-out. In Stage One, we will examine policies, use unstructured observations and undertake interviews with key health professionals to characterise the organisation and delivery of MDC. In Stage Two, observations of practice will continue, to deepen our understanding, and to inform two focused studies. The first will explore decision-making practices and the second will examine how staff engage with patients' needs; both studies involve interviews, to complement observation. As part of the study of supportive care, we will examine the implications of an introduction of patient-reported measures (PRMs) into care, adding surveys to interviews before and after PRMs roll-out. Data analysis will account for site-specific and cross-site issues using an adapted Qualitative Rapid Appraisal, Rigorous Analysis approach. Quantitative data from clinician surveys will be statistically analysed and triangulated with the related qualitative study findings.

Ethics and dissemination

For peer review only - http://bmjopen.bmj.com/site/about/guidelines.xhtml

Ethical approval was granted by South Eastern Sydney Local Health District Human Research Ethics Committee (no. 18/207). Findings will be shared with participating hospitals and widely disseminated through publications and presentations.

For peer review only

For peer review only - http://bmjopen.bmj.com/site/about/guidelines.xhtml

STRENGTHS AND LIMITATIONS OF THIS STUDY

- Multiple data types and sources are integrated to characterise MDC in different settings.
- A two-stage roll-out facilitates better targeting of observations and interviews in the second stage.
- A strategy of intensive data collection using multiple methods is used to ensure that site-specific data is interpreted within its local context.
- Findings will be based on a rigorous and extensive observational characterisation of Australian oncology outpatient services.

For peer review only

For peer review only - http://bmjopen.bmj.com/site/about/guidelines.xhtml

INTRODUCTION

In the developed world, invasive cancer is the leading cause of death.[1] Globally, the incidence of cancer is increasing;[2] in Australia, cancer incidence is projected to reach 150,000 new cases per year by 2020.[3] Survival rates are improving, with over 400,000 Australians living following a cancer diagnosis in the previous 5 years.[3] These trajectories frame cancer as a condition requiring medical and supportive care services from diagnosis through long-term survivorship.[4] Multidisciplinary care (MDC) is accepted as a best practice model of care provision for oncology services in Australia, and in advanced health systems globally.[5-9] MDC seeks to promote equitable, evidence-based care by a team that combines relevant expertise and facilitates active patient involvement.[6,10]

Work has been undertaken to translate MDC principles into practice; but significant challenges remain. Substantial evidence points to gaps between optimal evidence-based care and current care.[11-15] A review of research literature, national health sector reports and local cancer plans identified three key interconnected elements of MDC: diverse professional expertise; clinical decision-making; and, addressing patients' supportive care needs. The coordination of care binds these elements, as depicted in Figure 1.

<INSERT FIGURE 1. HERE>

Source: Authors' conceptualisation of MDC

Patient needs

In addition to medical treatment, cancer patients also need medical (e.g., pain relief) and non-medical supportive care (e.g., informational, practical, psychosocial). The challenges of addressing supportive needs are an ongoing concern in oncology.[16-18] Meeting these needs requires identification, prioritisation, treatment planning and timely access to relevant

For peer review only - http://bmjopen.bmj.com/site/about/guidelines.xhtml

professional expertise.[16-19] In general, identification of support needs in cancer care has not been systematic. Rather, routine care has relied on the clinical acumen of individual clinicians who identify needs during consultations and make appropriate referrals. Language spoken and level of health literacy also remain challenges in order to “pitch” information at the level most suitable for each patient. Routinely collecting information from patients, through Patient Reported Measures (PRMs), has been promoted as a mechanism to identify unmet supportive needs.[20-22] PRMs are planned to be introduced into routine cancer care in some of the outpatient clinics (OPCs) during the period of study, likely changing the management of patients' supportive needs.[23-25] This study focuses on the provider perspective; the patient's support networks are also important for addressing supportive needs, but these are beyond the study's scope.

Diverse professional expertise

MDC is overseen by a core team which, while varying by cancer type, usually consists of pathologists, radiologists, medical oncologists, radiation oncologists, surgeons, nurses and allied health professionals.[6,10] Other specialist expertise can also be part of the core team, or can be added as required (e.g., palliative care).[6] This approach is intended to utilise cross-disciplinary expertise to the fullest benefit of the patient and offer shared decision-making mechanisms for clinicians.[10,26] To develop an understanding of MDC we therefore need to harness the views of a diverse range of professionals and observe interprofessional collaboration in practice.

Decision-making

Multidisciplinary team meetings (MDTMs) are a fulcrum for the clinical coordination of MDC.[27,28] Research suggests that MDTM discussions influence diagnosis, staging and treatment planning.[29-31]It is important to understand the organisation of MDTMs and the resources required to support them. Studies reveal diversity in the organisation of MDTMs,

For peer review only

who is discussed and when, and the ways that clinicians engage to support clinical decision-making.[9] While MDTMs play a central role, clinicians make decisions outside MDTMs, without any consultation during routine care provision, or in consultation with other clinicians who may or may not be part of the team. In addition, decisions can be made at the meetings but evidence suggests that the recommendations are not always followed and in many meetings there can be disagreements in opinion. How these issues are managed with the patient are likely to be highly individualised. We also seek to understand this behaviour.

Coordinated care

Among the top priorities for coordination are: 1) timely and appropriate delivery of care across the patient's journey; and 2) ongoing screening of patients' supportive needs and appropriate care in response.[32] Public hospital OPCs are tasked with providing care for diverse types of cancer across the patient journey, from diagnosis, through treatment and into survivorship. The models of coordination employed may be non-uniform; hybrid approaches may be used within a setting (e.g., simultaneously offering tumour stream-specialised care coordination, with generalised care coordinators covering all others).[33,34] Sub-optimal coordination can lead to fragmented care, conflicting information provided to patients, impaired access to appropriate services and inefficient use of the time and energy of health professionals and patients.[19,35,36]

Research framework

The investigation described in this paper is the first major phase of field research undertaken by the newly established National Health and Medical Research Council (NHMRC) Centre for Research Excellence in Implementation Science in Oncology (CRE-ISO), administered by the Australian Institute for Health Innovation at Macquarie University. In healthcare, implementation science focuses on understanding processes and methods for supporting the uptake and integration of effective interventions to improve healthcare practices

For peer review only - http://bmjopen.bmj.com/site/about/guidelines.xhtml

and outcomes.[37] The research described here will characterise MDC provision in two Sydney metropolitan Local Health Districts (LHDs), government agencies responsible for managing and providing public health services within a specified geography, usually of approximately one million residents to provide a foundational understanding of the realities of oncology service provision. This understanding will inform subsequent phases of research undertaken by the Centre, some of which will focus on developing and testing tailored interventions to address identified evidence-practice gaps. Health economics studies will assess the potential costs and benefits of alternative improvement effort options to inform implementation, and evaluate the costs and benefits associated where implemented.

Critical to this study is engagement with the context of service provision and its complexity; of the “interacting and interdependent” components of a health service that comprise MDC provision in hospitals.[38] Multiple sources of data will be harvested and integrated, exploring the formalised processes and local practices undertaken within the interconnected networks of semi-autonomous professionals that together shape care provision.[39] Systems of care provision in these settings are rarely static, and opportunities will be taken to examine the uptake of new practices, such as the introduction of PRMs, to learn about the processes that inhibit or promote effective adoption. This research will also seek to identify generic barriers and facilitators that impede or promote best practice, as background information to assist with the design of future interventions, and health economics studies.

Research Aims (RA)

The study aims to:

RA1. Develop understanding of cancer services in OPCs by characterising the organisation and practice of MDC;

RA2. Explore, in-depth, selected key elements of MDC:

For peer review only - http://bmjopen.bmj.com/site/about/guidelines.xhtml

i. Clinical decision-making in MDC and how doctors engage with decision-making support mechanisms in practice; and

ii. How health professionals engage with patients' supportive needs before and immediately after the introduction of PRMs.

RA3. Identify general barriers to, and facilitators of, the provision of MDC.

METHODS & ANALYSIS

A mixed-methods research design will be adopted: largely comprised of a variety of qualitative methods, complimented by a single quantitative survey. Methods adapted from ethnography will be harnessed, to facilitate intensive and efficient data collection; rapid ethnography will be used to reduce the burden of research on organisations and participants while maintaining rigour and richness.[40,41] Careful thought has been given to the demands of multi-site research and we adopt recommended strategies used to "time-deepen",[42] enhance validity and maintain capacity to adequately engage with the complexities of the settings.

The methodological principles embedded in the design are: exploring everyday practices and the organisational relations that shape them, by observing participants at work in OPC settings; harnessing a plurality of participant perspectives, by interviewing professionals about their work; and generating rich descriptive accounts of MDC contexts by using analytical and interpretative approaches formulated for multi-site data.[43] A critical realist philosophical position is taken, which is underpinned by a realist ontological assumption and a critical epistemology.[44,45] From this position it is assumed that a given account may not perfectly reflect reality; rather, accounts may be contextually and structurally mediated, offering partial insight into complex phenomena.[46,47]

Design

For peer review only

Implementation will be staged. Prior to study commencement, profiles and structural descriptions of each hospital setting were developed, using publicly-available information. In *Stage One,* an overview of the organisation of practice and models of care will be derived through interviews with "Navigators" (e.g., OPC staff with an overview of care pathways such as Nurse Unit Managers or care coordinators), review of unpublished policies and procedures and unstructured observations of care provision. In *Stage Two*, we enrich the accounts of these settings through additional observation in OPCs. Together, these methods address the first research aim (RA1) and provide a foundation for the second (RA2) and third (RA3).

Stage Two also includes focused studies, undertaken in parallel. In Study 2.1, interviews with doctors and observations of cancer MDTMs provide insight into clinical decision-making practices in MDC and how doctors engage with decision-making support mechanisms (RA2.i). Engagement with patients' supportive needs will be explored in Study 2.2 (RA2.ii), using a two part, mixed-method design consisting of interviews with health professionals, observation of cancer MDTMs and a "clinician readiness" survey; all research elements will be performed before introduction of new PRMs, and repeated after they have been introduced.

Barriers and facilitators to the provision of MDC (RA3) will be elicited as part of the interviews in each study and enhanced by our observational studies in *Stages One* and *Two*. The study design is informed by the Consolidated Criteria for Reporting Qualitative Research.[48] The study will run for up to 23 months over 2019 and 2020.

Selection of sites and participants

The investigation will be undertaken within two metropolitan LHDs, each containing three participating public hospitals with OPCs. Collectively, the OPCs offer an extensive range of consultation, coordination, wellness and outpatient treatment services. Each LHD contains two large hospitals which provide an extensive range of services and a smaller hospital offering

For peer review only - http://bmjopen.bmj.com/site/about/guidelines.xhtml

a more limited range of services, closely linked with larger centres (e.g., including the provision of teleconferencing for meetings).

Sites and meetings: In *Stage One* all sites will be studied, to understand each hospital setting. In *Stage Two*, maximum variation sampling[49] will be used to allocate the 'continuing observation' sessions to participating sites. As there are differences in the services between sites (e.g. some hospitals offer a smaller range), observation sessions will be allocated to best capture the diverse range of practices and processes useful in the characterisation of MDC within and across sites. Purposive sampling will be used to select the MDTMs approached to participate. We will generate a sample comprising a range of tumour streams including common and rarer cancers. Study 2.1 will largely be conducted in one LHD, with study 2.2 in the other.

Participants: The participant inclusion criteria for interviews, surveys and health professional observations is generally broad: core medical staff (e.g., medical oncologists, surgeons, radiation oncologists); nursing staff (e.g., clinical nurse consultants, cancer care coordinators); and allied health staff (e.g., social workers, psychologists). However, participation in *Stage One* "Navigator interviews" will be restricted to staff with an overview of care pathways (e.g., cancer care coordinators, Nurse Unit Managers, managers of clinical streams). Similarly, in Study 2.2 the "clinician decision-making" interviews will be undertaken with specialist doctors (e.g. medical oncologists, surgeons, radiation oncologists) working in cancer services.

Recruitment: Information about the research including contact details and participant information sheets will be disseminated through email, flyers and posters, and through information sessions for target professional groups at each site. Snowball sampling will also be used; participants will be encouraged to discuss the project with colleagues. Letters will be sent to the Chairpersons of cancer MDTMs and to the authorities responsible for the OPCs, to

For peer review only - http://bmjopen.bmj.com/site/about/guidelines.xhtml

provide information about the research and request permission for the observations. These letters will include "Health Professional Information Sheets" which recipients will be encouraged to share with relevant colleagues as a component of pre-approval consultation.

Sample size ranges: The Study design features, presented in Table 1, summarise the stages of the research, the data collected in each stage and the target number of observations, interviews and surveys. The sample sizes designated for the interview components are guided by principles identified in reported research literature, as well as Malterud, Siersma and Guassora's (2016) model for generating "information power" in qualitative studies. The model accounts for the following factors when estimating a sample range: the study aim; specificity of the sample; the envisioned quality of the interview interaction; analysis strategy; and established theory.[50] The sample size allocated for the individual health professional observations reflects the narrow and specific focus of gaining a deep understanding of the roles these professionals play in MDC. The number of sessions proposed for the observations of the OPCs and MDTMs are comparable with similar qualitative studies[49] and are appropriate for multi-site ethnographic studies.[52]

Research Team: The investigation will be overseen by a team with extensive experience in health services and quality improvement research, including leaders in implementation science and oncology. Fieldwork will be guided by principal investigators with direct clinical and research experience in oncology service provision. Data will be collected by three researchers (non-clinicians) trained in qualitative, quantitative and mixed-methods investigation. Each of these researchers has fieldwork experience across a range of health services. Appropriately qualified health professionals who join the research team for *Stage Two* will be trained and supervised.

Table 1. Study design features

For peer review only - http://bmjopen.bmj.com/site/about/guidelines.xhtml

Phase/Stage	Text	Interviews	Survey	MDTM Observations	Undirected Observation Sessions*
**Stage One**					
**Stage One Multi-site characterisation**	Review of policies and procedures relevant to interview discussion	• Navigator (n=8-16)<br>• Decision-making (n=3-8)<br>• Supportive needs (n=4-10)	n/a	Cancer MDTMs (n=3-8)	Initial:<br>• OPCs (n=15-25)<br>• Individual (n=3-6)
**Stage Two**					
**Study 2.1 Decision-making**	Review of policies and procedures relevant to interview discussion	Decision-making (n=15-30)	n/a	Cancer MDTMs (n=3-8)	Ongoing:<br>• OPCs (n=15)<br>• Individual (n=7)
**Study 2.2. Supportive needs**		• Baseline (n=15-30)<br>• Follow-up (n=15-30)	• Baseline (n=30-50)<br>• Follow-up (n=30-50)	Cancer MDTMs (n=3-8)	
**Estimated totals**	n/a	60- 124	30-50	9-24	• OPCs (n=30-40)<br>• Individual (n=10)

Table key

	Undertaken at all sites
	Predominantly undertaken in LHD 1
	Predominantly undertaken in LHD 2
*	1 observation session= half day or 3-4hrs

Legend: MDTM=Multidisciplinary Team Meeting; OPC=cancer outpatient clinic.

* 1 observation session= half day or 3-4hrs

For peer review only - http://bmjopen.bmj.com/site/about/guidelines.xhtml

Data Collection

As part of protocol development, reviews of the literature were conducted to conceptualise and identify key issues in MDC provision and to scope the methodological scholarship relevant to the design. Publicly-available annual reports and cancer plans from each health district were analysed to generate a description of cancer services at each hospital. These profiles were used to map key areas for data collection. Implementation science approaches emphasise the importance of the local context of adoption and engagement with its stakeholders.[37-39] During this stage, input was sought from oncology management to ensure the local relevance of the research and the feasibility of the proposed methods.

Stage One. Multi-site characterisation study

Interviews: Semi-structured "Navigator" interviews will be undertaken with professionals who have overview roles (e.g., cancer care coordinator), oriented by care pathway maps, to establish the models of care and processes involved in the patient journey (n=8-16).[53,54] The care pathway maps were derived from the stages of MDC depicted by Fennell, Das, Clauser, Petrelli and Salner's (2010),[55] focused on the diagnosis and OPC treatment stages (Figure 2). Through the interviews, maps will be reworked to reflect the differing trajectories followed by different: a) tumour streams (accounting for tumour-specific services); and b) risk categories and complexity. Where interviewees refer to specific policies and procedures, documentation will be formally requested to help situate the discussion within its policy context. Interviews are expected to take approximately 40 minutes and will be audio-recorded and transcribed.

Observations: Three types of observations will be made in the research:

- Unstructured observations of OPCs will provide an understanding of the real-world, routine organisation and actual delivery of MDC in these settings (e.g., we will observe *ad hoc* and

For peer review only - http://bmjopen.bmj.com/site/about/guidelines.xhtml

planned collaboration between professionals from differing disciplines during the care delivery process; n=15-25).

- Observations of individual health professionals will capture the key activities undertaken, to understand how their work contributes to the provision of MDC (n=3-6 individual health professionals observed for half or all of their day).

- Observations of MDTMs will be conducted using a semi-structured template (Figure 4) informed by previous MDTM observational research (n=3-8 meetings).[27, 56] Fieldnotes will record observations of: professional groups in attendance; specific roles; support provided to administer the meeting (i.e. technology); organisation of cases; clinical decision-making; patient-centred care, and; organisation of follow-up.

All observations will be recorded in hand-written fieldnotes which will be used to generate rich accounts of care.

<INSERT FIGURE 2. HERE>

Source: Fennell et al. (2010)[55] (adapted from Zapka et al., 2003)[57]

Transition to Stage Two: During *Stage One*, we will pilot elements of the clinician interviews about decision-making (n=3-8) and patients' supportive needs (n=4-10) to be undertaken in *Stage Two,* and interview prompts will be refined. Where eligible health professionals indicate interest in joining the research team for *Stage Two* fieldwork, they will be briefed and provided with necessary training and support. Modifications to methods and the research team composition will be submitted for ethical approval, if required.

For peer review only - http://bmjopen.bmj.com/site/about/guidelines.xhtml

<INSERT FIGURE 3: HERE>

Source: Developed by authors based on Harris et al (2016)[56] and Rankin et al. (2017)[27]

Stage Two. MDC Studies

This stage will enrich accounts of MDC provision via “ongoing characterisation” (i.e. n=3-6 further observations of individual health and n=15 unstructured observations of OPCs), at the same time contextualising the focused studies detailed below.

Study 2.1 Clinical decision-making

Interviews: Doctors will participate in semi-structured interviews that explore their decision-making practices and their engagement with support mechanisms (n=15-30). Topics will include their perceptions of decision-making mechanisms, the decision-making support available to them, their own decision-making practices and their use of MDTMs at key points in the patient journey. A conceptual map of the patient journey (Figure 5) will be used to orient the interview, with questions tailored to each stage. Barriers to and drivers of efficient clinical decision-making will be explored. Interviews are expected to take approximately 40 minutes and will be audio-recorded and transcribed.

<INSERT FIGURE 4. HERE>

Source: Authors’ adaption of Fennell et al. (2010)[55]

For peer review only - http://bmjopen.bmj.com/site/about/guidelines.xhtml

Observation of MDTMs: Meetings will be observed using a semi-structured template (Figure 4), focussing on the "clinical decision-making" component (n=3-8). Observational data will capture information about this decision-making mechanism and contextualise interviews.

Study 2.2 Patients' supportive needs

Interviews: Prior to introduction of PRMs, semi-structured interviews will be conducted with health professionals about how they engage with patients' supportive needs in their current practice (n=15-30). Topics will include: models of care; their individual role; identification and screening; management; and any barriers and facilitators experienced in identifying and responding to supportive needs. The interviews will also explore preparations for and expectations of PRMs.

Following the adoption of PRMs, health professionals will again participate in semi-structured interviews (n=15-30). These will focus on the adoption and implications of PRMs in relation to the topics addressed in pre-introduction interviews. Pre- and post-introduction interviews are expected to take approximately 40 minutes and will be audio-recorded and transcribed.

Surveys: An eight-item clinician readiness survey (Willis et al. 2009)[58] will be completed by health professionals before and after the adoption of PRMs (n=30-50 in each phase). The survey will assess the current level of preparedness, and changes over time. This survey will take approximately five minutes to complete and will be performed as part of the interviews and will also be distributed more widely to achieve the desired sample size.

Observation of MDTMs: Meetings will be observed using a semi-structured template (Figure 4), focussing on the "patient-centred care & supportive care needs" component (n=3-

For peer review only - http://bmjopen.bmj.com/site/about/guidelines.xhtml

8). Data will capture information about engagement with supportive needs in MDTMs and contextualise the interviews.

Data Analysis

Interview data: Basic descriptive data collected during interviews (e.g. listing of team members involved in different points of the patient journey) will be collated across interviews then categorised and organised within a matrix. The discursive data will be thematically analysed, oriented by an inductive approach.[59] Where process maps are used (e.g. navigator and decision-making interviews), these will frame the analysis. Constant comparative analysis will be applied to interpret data gathered before and after the introduction of PRMs. This strategy accounts for the cross-site, multiple-source nature of the data. Our approach for making sense of cross-site data is informed by the "Qualitative Rapid Appraisal, Rigorous Analysis" methodology developed by Phillips et al. (2014).[41] First, all "like" data (e.g., all interview data) is analysed at a site, followed by intra-site analysis of all data from one OPC. Inter-site analysis is then employed to consider the data across the sites.[41]

Observational data: Fieldnotes will be indexed to identify the specific setting/MDTM meeting, key processes and events and study focus. The notes will be integrated within hospital cancer service profiles to elaborate descriptions of OPC settings (i.e., the organisational features of care). Fieldnotes from MDTM and OPC observations will then be developed into accounts that contextualise data collected in the focused studies. Data collected from individual health professional observations will be developed into an account of the role.

Clinician readiness survey data: Analysis will include a repeated-measures ANOVA to test for differences in clinician attitudes before and after PRMS roll-out. Subgroup analyses will test for differences in attitudes between different demographic and clinical subgroups. The quantitative data will be used to complement the interview data on this topic. Based on known effect sizes from results of the Willis et al. (2009)[58] paper using the Outcome Measurement

For peer review only - http://bmjopen.bmj.com/site/about/guidelines.xhtml

Questionnaire to assess change in response to training, using a power of 0.8 and alpha of 0.05, the minimum sample size was estimated as n=32 participants (i.e., to detect a change in mean scale score from 4.09 pre- to 4.49 post-introduction, with a standard deviation of 0.57).

Measurement of outcomes

For the qualitatively-driven research, assessment of the outcomes involves judgement of the adequacy with which the research aims were addressed. This is largely reliant on the quality of the data collected and the rigour of its analysis. Sufficiently detailed data are needed from a range of sources to produce rich descriptions of MDC practice, including its diversity. Fieldwork and analytic strategies should allow findings to be situated within OPC contexts as well as revealing points of connection and contradiction between sites. Characterisation of MDC provision will be achieved through credible, adequately complex and well-grounded accounts. Multi-method data collection (including a quantitative component) allows for consideration of various sources of input, and triangulation; a team-based approach to analysis will facilitate ongoing reflection and monitoring.

For peer review only

For peer review only - http://bmjopen.bmj.com/site/about/guidelines.xhtml

ETHICS & DISSEMINATION

Ethical approval was granted by South Eastern Sydney LHD Human Research Ethics Committee (#18-207). Informed written consent will be obtained from interviewees and from health professionals who are individually observed. Interview and observation arrangements will be made with health professionals at least one week in advance to ensure they have time to prepare. Participants may withdraw, without justification, up to one-week post interview/observation or whenever the data is de-identified.

Posters will be displayed, and information sheets will be available during observational sessions so that those present can raise concerns or decline participation. This can be done at any time up until the observational data is collected. The research team will not collect any information about patients and, guided by health professionals, will minimise exposure to direct patient interactions. Researchers will answer questions posed about their presence and provide information sheets that contain ethics committee details. In the event of participants experiencing discomfort or distress arising from the research, researchers will facilitate access to appropriate support services.

Data storage and protection: Data will be stored on a password-protected computer and on a secure University server, accessible only to the research team. Data will be de-identified and separated from participant identifiers. Research records will be retained for at least five years post-study completion or last publication, in accordance with Section 2.1.1 of the Australian Code for the Conduct of Responsible Research (2007)[60] before secure destruction.

Dissemination: Findings will be disseminated to academics, professionals and the public through publications and presentations. Tailored reports will be provided to participating hospitals and presentations given to participating professional groups.

For peer review only - http://bmjopen.bmj.com/site/about/guidelines.xhtml

Patient and Public Involvement Statement

The coordinating research institution routinely consults with patients, their representatives and the general public, to ensure that adequate input is secured for research programs; a key partner is the Consumers Health Forum of Australia. This specific project was developed without detailed patient involvement; patients were not invited to comment on the study design or to the writing or editing of this protocol. This project concerns provider perspectives of care delivery by OPCs. Patient-centred studies are anticipated for subsequent phases of the research program of which this study is a part.

For peer review only

For peer review only - http://bmjopen.bmj.com/site/about/guidelines.xhtml

IMPACT & SIGNIFICANCE

The research seeks to capture a diverse range of practices and processes useful in the characterisation of MDC, with attention to site-specific nuances that may be relevant to future improvement efforts or health economic studies. The accumulative characterisation will be combined with focused, in-depth studies of clinical decision-making and processes for meeting patients' supportive care needs. The research involves rigorous and extensive observations of Australian public oncology OPCs and MDTMs; it offers a substantial opportunity for the generation of new and critical insights. The findings will serve as a foundation for the design of studies to address identified evidence-practice gaps in cancer care.

For peer review only

For peer review only - http://bmjopen.bmj.com/site/about/guidelines.xhtml

References

1. Jemal A, Bray F, Center MM, et al. Global cancer statistics. *CA Cancer J Clin* 2011;61(2):69-90. doi:10.3322/caac.20107

2. International Agency for Research on Cancer. GLOBOCAN 2018 Global Cancer Observatory, 2018.

3. Australian Institute of Health and Welfare. Cancer incidence projections, Australia 2011 to 2020. Canberra, 2012.

4. Australian Institute of Health and Welfare. Cancer in Australia Canberra, 2017.

5. American College of Surgeons: Commission on Cancer. Cancer Program Standards: Ensuring Patient-Centered Care Chicago, IL: American College of Surgeons, 2016.

6. Cancer Australia. All about multidisciplinary care. Available: https://canceraustralia.gov.au/clinical-best-practice/multidisciplinary-care/all-about-multidisciplinary-care (accessed 20 July 2018).

7. Jiang H. Patients should surely be included in multidisciplinary team meetings in China healthcare. Response to Thornton, S (2015) Time to review utility of multidisciplinary team meetings. *BMJ* 2015;351:5295 doi: 10.1136/bmj.h5295

8. Pan C-C, Kung P-T, Wang Y-H, et al. Effects of multidisciplinary team care on the survival of patients with different stages of non-small cell lung cancer: A national cohort study. *PloS One* 2015;10(5):e0126547-e47. doi: 10.1371/journal.pone.0126547

9. Saini KS, Taylor C, Ramirez AJ, et al. Role of the multidisciplinary team in breast cancer management: results from a large international survey involving 39 countries. *Ann Oncol* 2012;23(4):853-59. doi: 10.1093/annonc/mdr352

10. Zorbas H, Barraclough B, Rainbird K, et al. Multidisciplinary care for women with early breast cancer in the Australian context: What does it mean? *Med J Aust* 2003;179(10):528-31.

11. Barton MB, Jacob S, Shafiq J, et al. Estimating the demand for radiotherapy from the evidence: A review of changes from 2003 to 2012. *Radiol Oncol* 2014;112(1):140-44. doi: 10.1016/j.radonc.2014.03.024

12. Batumalai V, Shafiq J, Gabriel G, et al. Impact of radiotherapy underutilisation measured by survival shortfall, years of potential life lost and disability-adjusted life years lost in New South Wales, Australia. *Radiol Oncol* 2018;129(2):191-95. doi: 10.1016/j.radonc.2018.06.026

For peer review only - http://bmjopen.bmj.com/site/about/guidelines.xhtml

13. Delaney GP, Barton MB. Evidence-based Estimates of the Demand for Radiotherapy. *Clin Oncol* 2015;27(2):70-76. doi: 10.1016/j.clon.2014.10.005

14. Hanna TP, Shafiq J, Delaney GP, et al. The population benefit of evidence-based radiotherapy: 5-Year local control and overall survival benefits. *Radiol Oncol* 2018;126(2):191-97. doi: 10.1016/j.radonc.2017.11.004

15. Telli ML, Jensen KC, Vinayak S, et al. Phase II Study of Gemcitabine, Carboplatin, and Iniparib As Neoadjuvant Therapy for Triple-Negative and BRCA1/2 Mutation–Associated Breast Cancer With Assessment of a Tumor-Based Measure of Genomic Instability: PrECOG 0105. *J Clin Oncol* 2015;33(17):1895-901. doi: 10.1200/jco.2014.57.0085

16. Dilworth S, Higgins I, Parker V, et al. Patient and health professional's perceived barriers to the delivery of psychosocial care to adults with cancer: A systematic review. *Psychooncology* 2014;23(6):601-12. doi:10.1002/pon.3474

17. Rankin NM, Barron JA, Lane LG, et al. Psychosocial oncology services in New South Wales. *Aust Health Rev* 2011;35(2):156-63. doi: 10.1071/AH08730

18. Walsh J, Young JM, Harrison JD, et al. What is important in cancer care coordination? A qualitative investigation. *Eur J Cancer Care* 2011;20(2):220-27. doi:10.1111/j.1365-2354.2010.01187.x

19. Walsh J, Harrison JD, Young JM, et al. What are the current barriers to effective cancer care coordination? A qualitative study. *BMC Health Serv Res* 2010;10(1):132. doi: 10.1186/1472-6963-10-132

20. Basch E, Deal AM, Kris MG, et al. Symptom Monitoring With Patient-Reported Outcomes During Routine Cancer Treatment: A Randomized Controlled Trial. *J Clin Oncol* 2016;34(6):557-65. doi: 10.1200/jco.2015.63.0830

21. Luckett T, Butow PN, King MT. Improving patient outcomes through the routine use of patient-reported data in cancer clinics: Future directions. *Psychooncology* 2009;18(11):1129-38.doi:10.1002/pon.1545

22. Williams K, Sansoni J, Morris D, et al. Patient-reported outcome measures: literature review. Australian Health Services Research Institute, University of Wollongong Australia, 2016.

For peer review only - http://bmjopen.bmj.com/site/about/guidelines.xhtml

23. Cancer Institute NSW. Patient-reported measures (PRMs) 2018. Available: https://www.cancer.nsw.gov.au/how-we-help/quality-improvement/patient-reported-measures (accessed 30 August 2018).

24. Girgis A, Delaney GP, Miller AA. Utilising ehealth to support survivorship care. *Cancer Forum* 2015; 39(2):86-89.

25. Girgis A, Durcinoska I, Levesque JV, et al. eHealth System for Collecting and Utilizing Patient Reported Outcome Measures for Personalized Treatment and Care (PROMPT-Care) Among Cancer Patients: Mixed Methods Approach to Evaluate Feasibility and Acceptability. *J Med Internet Res* 2017;19(10):e330. doi:10.2196/jmir.8360

26. National Breast Cancer Centre. Multidisciplinary Care (MDC) in Australia: A National Demons tration Project in Breast Cancer. Camperdown, NSW: National Breast Cancer Centre, 2003.

27. Rankin NM, Lai M, Miller D, et al. Cancer multidisciplinary team meetings in practice: Results from a multi-institutional quantitative survey and implications for policy change. *Asia Pac J Clin Oncol* 2018;14(1):74-83. doi:10.1111/ajco.12765

28. Wilcoxon H, Luxford K, Saunders C, et al. Multidisciplinary cancer care in Australia: A national audit highlights gaps in care and medico-legal risk for clinicians. *Asia Pac J Clin Oncol* 2011;7(1):34-40. doi:10.1111/j.1743-7563.2010.01369.x

29. Brunner M, Gore SM, Read RL, et al. Head and neck multidisciplinary team meetings: Effect on patient management. *Head Neck* 2015;37(7):1046-50. doi: doi:10.1002/hed.23709

30. Davies AR, Deans DAC, Penman I, et al. The multidisciplinary team meeting improves staging accuracy and treatment selection for gastro-esophageal cancer. *Dis Esophagus* 2006;19(6):496-503. doi: 10.1111/j.1442-2050.2006.00629.x

31. Friedland PL, Bozic B, Dewar J, et al. Impact of multidisciplinary team management in head and neck cancer patients. *Br J Cancer* 2011;104:1246. doi: 10.1038/bjc.2011.92

32. Shaw T, York S, White K, et al. Defining success factors to describe coordinated care in cancer. *Transl Behav Med* 2018;8(3):357-65. doi: 10.1093/tbm/iby022

For peer review only - http://bmjopen.bmj.com/site/about/guidelines.xhtml

33. Eicher MRE, Marquard S, Aebi S. A nurse is a nurse? A systematic review of the effectiveness of specialised nursing in breast cancer. *Eur J Cancer* 2006;42(18):3117-26. doi: 10.1016/j.ejca.2006.07.007

34. Porter-Steele J, Tjondronegoro D, Seib C, et al. 'Not one size fits all': A brief review of models of care for women with breast cancer in Australia. *Cancer Forum* 2017;41(1):13-19.

35. Kripalani S, Jackson AT, Schnipper JL, et al. Transitions of Care at Hospital Discharge. *J Hosp Med* 2007;5:314-23. doi:10.1002/jhm.228

36. Weaver SJ, Jacobsen PB. Cancer care coordination: opportunities for healthcare delivery research. *Transl Behav Med* 2018;8(3):503-08. doi: 10.1093/tbm/ibx079

37. Rapport F, Clay-Williams R, Churruca K, et al. The struggle of translating science into action: Foundational concepts of implementation science. *J Eval Clin Pract* 2018;24(1):117-26. doi:10.1111/jep.12741

38. Braithwaite J, Marks D, Taylor N. Harnessing implementation science to improve care quality and patient safety: a systematic review of targeted literature. *Int J Qual Health Care* 2014;26(3):321-29. doi: 10.1093/intqhc/mzu047

39. Braithwaite J, Churruca K, Long JC, et al. When complexity science meets implementation science: a theoretical and empirical analysis of systems change. *BMC Med* 2018;16(1):63. doi: 10.1186/s12916-018-1057-z

40. Handwerker W. Quick Ethnography. Walnut Creek, CA: Altamira Press 2001.

41. Phillips CB, Dwan K, Hepworth J, et al. Using qualitative mixed methods to study small health care organizations while maximising trustworthiness and authenticity. *BMC Health Serv Res* 2014;14(1):559. doi: 10.1186/s12913-014-0559-4

42. Millen D. Rapid ethnography: Time deepening strategies for HCI field research. Proceedings of the 3rd conference on Designing interactive systems: processes, practices, methods, and techniques. New York City, New York, USA: ACM, 2000:280-86.

43. Charlesworth S, Baines D. Understanding the negotiation of paid and unpaid care work in community services in cross-national perspective: The contribution of a rapid ethnographic approach. *J Fam Stud* 2015;21(1):7-21. doi: 10.1080/13229400.2015.1010263

For peer review only - http://bmjopen.bmj.com/site/about/guidelines.xhtml

For peer review only

44. Harper D. Choosing a qualitative research method. In: Harper D, Thompson AR, eds. Qualitative Research Methods in Mental Health and Psychotherapy: A Guide for Students and Practitioners Hoboken, NJ: Wiley-Blackwell 2011.

45. Bentall DPR. The medicalisation of misery: A critical realist analysis of the concept of depression. *J Ment Health* 1999;8(3):261-74. doi: 10.1080/09638239917427

46. Harper D, Thompson AR. Qualitative Research Methods in Mental Health and Psychotherapy: A Guide for Students and Practitioners. Hoboken, NJ: Wiley-Blackwell 2011.

47. Willig C. Introducing Qualitative Research in Psychology. Maidenhead, UK: McGraw-Hill Education 2013.

48. Tong A, Sainsbury P, Craig J. Consolidated criteria for reporting qualitative research (COREQ): a 32-item checklist for interviews and focus groups. *Int J Qual Health Care* 2007;19(6):349-57. doi: 10.1093/intqhc/mzm042

49. Palinkas LA, Horwitz SM, Green CA, et al. Purposeful sampling for qualitative data collection and analysis in mixed method implementation research. *Adm Policy Ment Health* 2015;42(5):533-44. doi: 10.1007/s10488-013-0528-y

50. Malterud K, Siersma VD, Guassora AD. Sample Size in Qualitative Interview Studies: Guided by Information Power. *Qual Health Res* 2015;26(13):1753-60. doi: 10.1177/1049732315617444

51. Bridges J, Hughes J, Farrington N, et al. Cancer treatment decision-making processes for older patients with complex needs: A qualitative study. *BMJ Open* 2015;5(12):e009674. doi: 10.1136/bmjopen-2015-009674

52. Renedo A, Marston C. Developing patient-centred care: An ethnographic study of patient perceptions and influence on quality improvement. *BMC Health Serv Res* 2015;15(1):122. doi: 10.1186/s12913-015-0770-y

53. Lichtner V, Dowding D, Allcock N, et al. The assessment and management of pain in patients with dementia in hospital settings: A multi-case exploratory study from a decision making perspective. *BMC Health Serv Res* 2016;16(1):427. doi: 10.1186/s12913-016-1690-1

54. Trebble TM, Hansi N, Hydes T, et al. Process mapping the patient journey: an introduction. *BMJ* 2010;341 doi: 10.1136/bmj.c4078

For peer review only

55. Fennell ML, Prabhu Das I, Clauser S, et al. The Organization of Multidisciplinary Care Teams: Modeling Internal and External Influences on Cancer Care Quality. *JNCI Monographs* 2010;(40):72-80. doi: 10.1093/jncimonographs/lgq010

56. Harris J, Taylor C, Sevdalis N, et al. Development and testing of the cancer multidisciplinary team meeting observational tool (MDT-MOT). *Int J Qual Health Care* 2016;28(3):332-38. doi: 10.1093/intqhc/mzw030

57. Zapka JG, Taplin SH, Solberg LI, et al. A Framework for Improving the Quality of Cancer Care: The Case of Breast and Cervical Cancer Screening. *Cancer Epidemiol Biomarkers Prev* 2003;12(1):4-13.

58. Willis A, Deane FP, Coombs T. Improving clinicians' attitudes toward providing feedback on routine outcome assessments. *Int J Ment Health Nurs* 2009;18(3):211-15. doi:10.1111/j.1447-0349.2009.00596.x

59. Joffe H. Thematic Analysis. In: Harper D, Thompson AR, eds. Qualitative Research Methods in Mental Health and Psychotherapy: A Guide for Students and Practitioners. Chichester: John Wiley & Sons, Ltd. 2011.

60. National Health Medical Research Council. Australian Code for the Responsible Conduct of Research: National Health and Medical Research Council, the Australian Research Council and Universities Australia, 2007.

For peer review only

For peer review only - http://bmjopen.bmj.com/site/about/guidelines.xhtml

Declarations

Authors' contributions:

JB, GD, WL, RW, DC, IO, JK and JW led the conceptualisation of the work and the development of the design was led by JB, GD, WL, GA, BNGE, YT, MB, KL, RC and TW. With guidance from the other authors, BNGE wrote the first draft and all authors contributed to revising key content to develop the final version. All authors have seen and approve of the final manuscript.

Funding statement:

JB receives funding from a variety of sources to support this work including NHMRC grant APP9100002.

Competing interests statement

None to declare

For peer review only

For peer review only - http://bmjopen.bmj.com/site/about/guidelines.xhtml

List of tables and figures

Figure 1. Key elements of MDC

Figure 2. MDC interventions at transitions in cancer care

Figure 3. MDTM data collection template

Figure 4. Mapping of points of diagnosis and treatment decision-making in MDC

For peer review only

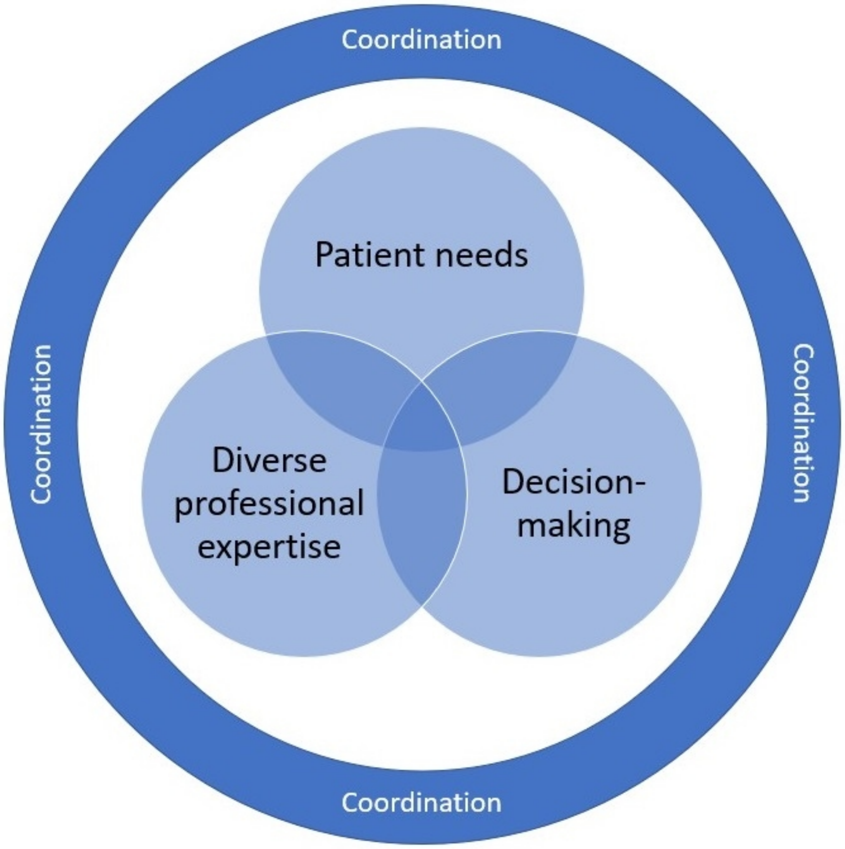

Source: Authors' conceptualisation of MDC

66x58mm (300 x 300 DPI)

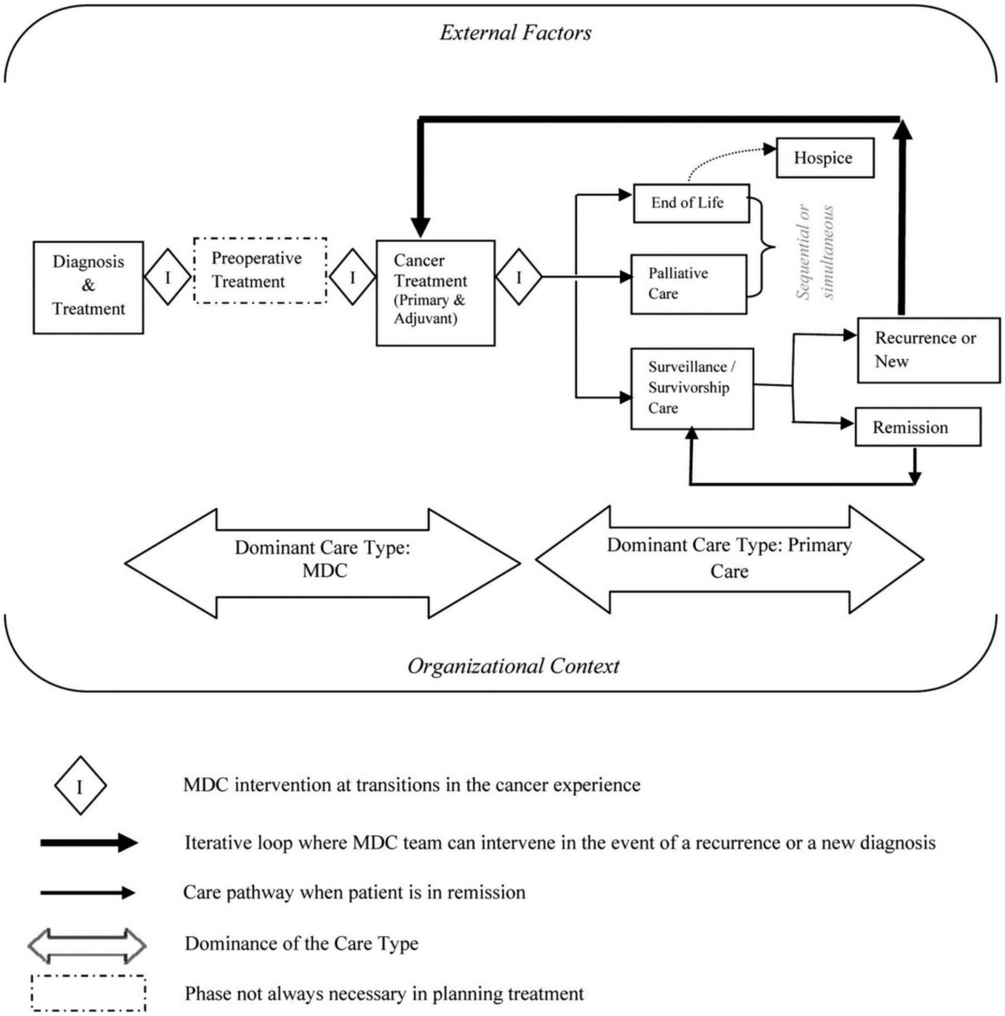

Source: Fennell et al. (2010)55 (adapted from Zapka et al., 2003)57

188x190mm (300 x 300 DPI)

MDTM & Hospital:<br>Date:	Notes
Attendance of health professional groups represented at MDT meetings by various health professional groups	
Chairperson and any meeting specific roles allocated	
Technological, equipment and administrative support in MDT meetings	
Organisation of the presentation of cases including<br>• The order<br>• Any prioritisation strategies<br>• Any patient inclusion criteria for meetings	
Clinical decision-making<br>• Discipline of professional input<br>• Types of information considered<br>• Evidence and/ or research incorporated into meetings	
Patient-centred care & Supportive care needs	
Organisation of any follow up tasks	

Source: Developed by authors based on Harris et al (2016)56 and Rankin et al. (2017)27

108x60mm (300 x 300 DPI)

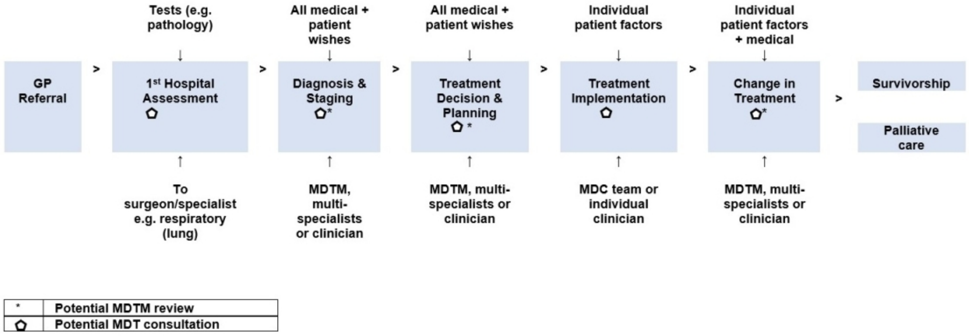

Source: Authors' adaption of Fennell et al. (2010)55

120x46mm (300 x 300 DPI)

COREQ (COnsolidated criteria for REporting Qualitative research) Checklist

A checklist of items that should be included in reports of qualitative research. You must report the page number in your manuscript where you consider each of the items listed in this checklist. If you have not included this information, either revise your manuscript accordingly before submitting or note N/A.

Topic	Item No.	Guide Questions/Description	Reported on Page No.
**Domain 1: Research team and reflexivity**			
Personal characteristics			
Interviewer/facilitator	1	Which author/s conducted the interview or focus group?	
Credentials	2	What were the researcher's credentials? E.g. PhD, MD	
Occupation	3	What was their occupation at the time of the study?	
Gender	4	Was the researcher male or female?	
Experience and training	5	What experience or training did the researcher have?	
Relationship with participants			
Relationship established	6	Was a relationship established prior to study commencement?	
Participant knowledge of the interviewer	7	What did the participants know about the researcher? e.g. personal goals, reasons for doing the research	
Interviewer characteristics	8	What characteristics were reported about the inter viewer/facilitator? e.g. Bias, assumptions, reasons and interests in the research topic	
**Domain 2: Study design**			
Theoretical framework			
Methodological orientation and Theory	9	What methodological orientation was stated to underpin the study? e.g. grounded theory, discourse analysis, ethnography, phenomenology, content analysis	
Participant selection			
Sampling	10	How were participants selected? e.g. purposive, convenience, consecutive, snowball	
Method of approach	11	How were participants approached? e.g. face-to-face, telephone, mail, email	
Sample size	12	How many participants were in the study?	
Non-participation	13	How many people refused to participate or dropped out? Reasons?	
Setting			
Setting of data collection	14	Where was the data collected? e.g. home, clinic, workplace	
Presence of non-participants	15	Was anyone else present besides the participants and researchers?	
Description of sample	16	What are the important characteristics of the sample? e.g. demographic data, date	
Data collection			
Interview guide	17	Were questions, prompts, guides provided by the authors? Was it pilot tested?	
Repeat interviews	18	Were repeat inter views carried out? If yes, how many?	
Audio/visual recording	19	Did the research use audio or visual recording to collect the data?	
Field notes	20	Were field notes made during and/or after the inter view or focus group?	
Duration	21	What was the duration of the inter views or focus group?	
Data saturation	22	Was data saturation discussed?	
Transcripts returned	23	Were transcripts returned to participants for comment and/or	

For peer review only

Topic	Item No.	Guide Questions/Description	Reported on Page No.
		correction?	
**Domain 3: analysis and findings**			
Data analysis			
Number of data coders	24	How many data coders coded the data?	
Description of the coding tree	25	Did authors provide a description of the coding tree?	
Derivation of themes	26	Were themes identified in advance or derived from the data?	
Software	27	What software, if applicable, was used to manage the data?	
Participant checking	28	Did participants provide feedback on the findings?	
Reporting			
Quotations presented	29	Were participant quotations presented to illustrate the themes/findings? Was each quotation identified? e.g. participant number	
Data and findings consistent	30	Was there consistency between the data presented and the findings?	
Clarity of major themes	31	Were major themes clearly presented in the findings?	
Clarity of minor themes	32	Is there a description of diverse cases or discussion of minor themes?	

Developed from: Tong A, Sainsbury P, Craig J. Consolidated criteria for reporting qualitative research (COREQ): a 32-item checklist for interviews and focus groups. *International Journal for Quality in Health Care*. 2007. Volume 19, Number 6: pp. 349 – 357

Once you have completed this checklist, please save a copy and upload it as part of your submission. DO NOT include this checklist as part of the main manuscript document. It must be uploaded as a separate file.

For peer review only

BMJ Open

What is Multidisciplinary Cancer Care Like in Practice? A protocol for a mixed-method study to characterise ambulatory oncology services in the Australian public sector

Journal:	*BMJ Open*
Manuscript ID	bmjopen-2019-031179.R1
Article Type:	Protocol
Date Submitted by the Author:	17-Jul-2019
Complete List of Authors:	Nic Giolla Easpaig, Bróna; Macquarie University, Australian Institute of Health Innovation<br>Arnolda, Gaston; Macquarie University, Australian Institute of Health Innovation<br>Tran, Yvonne ; Macquarie University, Australian Institute of Health Innovation<br>Bierbaum, Mia; Macquarie University, Australian Institute of Health Innovation<br>Lamprell, Klay; Macquarie University, Australian Institute of Health Innovation<br>Delaney, Geoffrey; Liverpool Cancer Therapy Centre; University of New South Wales South Western Sydney Clinical School<br>Liauw, Winston; Saint George Hospital Saint George Cancer Care Centre; University of New South Wales, St George Hospital Clinical School<br>Chittajallu, Renuka; Macquarie University, Australian Institute of Health Innovation<br>Winata, Teresa; Macquarie University, Australian Institute of Health Innovation; Griffith University,<br>Ward, Robyn; University of Sydney, Faculty of Medicine and Health; University of New South Wales, Prince of Wales Clinical School<br>Currow, David ; Flinders University, College of Medicine and Public Health; University of Technology Sydney, Faculty of Health<br>Olver, Ian; University of South Australia, Division of Health Sciences<br>Karnon, Jonathan; Flinders University, College of Medicine and Public Health<br>Westbrook, Johanna; Macquarie University, Australian Institute of Health Innovation<br>Braithwaite, Jeffrey; Macquarie University, Australian Institute of Health Innovation
**Primary Subject Heading**:	Health services research
Secondary Subject Heading:	Oncology, Health services research, Patient-centred medicine, Health economics, Evidence based practice
Keywords:	cancer, QUALITATIVE RESEARCH, multi-site, ONCOLOGY, outpatient clinic

SCHOLARONE™
Manuscripts

Title: What is Multidisciplinary Cancer Care Like in Practice? A protocol for a mixed-method study to characterise ambulatory oncology services in the Australian public sector

Authors:

1. Nic Giolla Easpaig, Bróna (Corresponding author)

   Postal Address: Australian Institute of Health Innovation, Macquarie University

   Level 6, 75 Talavera Road, Sydney, NSW 2109, Australia

   Email: brona.nicgiollaeaspaig@mq.edu.au

   Phone number: +61 2 9850 2400

2. Arnolda, Gaston

   Australian Institute of Health Innovation, Macquarie University, Sydney, NSW, Australia

3. Tran, Yvonne

   Australian Institute of Health Innovation, Macquarie University, Sydney, NSW, Australia

4. Bierbaum, Mia

   Australian Institute of Health Innovation, Macquarie University, Sydney, NSW, Australia

5. Lamprell, Klay,

   Australian Institute of Health Innovation, Macquarie University, Sydney, NSW, Australia

6. Delaney, Geoffrey, P.

   i. Liverpool Cancer Therapy Centre, Liverpool, NSW, Australia

For peer review only

For peer review only - http://bmjopen.bmj.com/site/about/guidelines.xhtml

ii. South Western Sydney Clinical School, University of New South Wales, Liverpool, NSW, Australia

7. Liauw, Winston,

i. St. George Cancer Care Centre, St. George Hospital, Kogarah, NSW, Australia

ii. St. George Hospital Clinical School, University of New South Wales, Sydney, NSW, Australia

8. Chittajallu, Renuka

Australian Institute of Health Innovation, Macquarie University, Sydney, NSW, Australia

9. Winata, Teresa

Australian Institute of Health Innovation, Macquarie University, Sydney, NSW, Australia

10. Ward, Robyn, L.

i. Faculty of Medicine and Health, The University of Sydney, Sydney, NSW, Australia

ii. Prince of Wales Clinical School, University of New South Wales, Sydney, NSW, Australia

11. Currow, David

i. College of Medicine and Public Health, Flinders University, Adelaide, SA, Australia

ii. Faculty of Health, University of Technology Sydney, Sydney, NSW, Australia

12. Olver, Ian,

Division of Health Sciences, University of South Australia, Adelaide, SA, Australia

13. Karnon, Jon,

College of Medicine and Public Health, Flinders University, Adelaide, SA, Australia

For peer review only

14. Westbrook, Johanna

Australian Institute of Health Innovation, Macquarie University, Sydney, NSW, Australia

15. Braithwaite, Jeffrey

Australian Institute of Health Innovation, Macquarie University, Sydney, NSW, Australia

For peer review only

Word count: *4,359* (excluding title page, abstract, references, figures and tables)

Keywords: qualitative research; multi-site; oncology; cancer; outpatient clinic

For peer review only - http://bmjopen.bmj.com/site/about/guidelines.xhtml

ABSTRACT

Introduction

An understanding of the real-world provision of oncology outpatient services can help maintain service quality in the face of escalating demand and tight budgets, by informing the design of interventions that improve the effectiveness or efficiency of provision. The aims of this study are threefold. First, to develop an understanding of cancer services in outpatient clinics by characterising the organisation and practice of multidisciplinary care (MDC). Second, to explore the key areas of: a) clinical decision-making; and b) engagement with patients' supportive needs. Third, to identify barriers to, and facilitators of, the delivery of quality care in these settings.

Methods and analysis

A suite of mixed-methods studies will be implemented at six hospitals providing cancer outpatient clinics (OPCs), with a staged roll-out. In Stage One, we will examine policies, use unstructured observations and undertake interviews with key health professionals to characterise the organisation and delivery of MDC. In Stage Two, observations of practice will continue, to deepen our understanding, and to inform two focused studies. The first will explore decision-making practices and the second will examine how staff engage with patients' needs; both studies involve interviews, to complement observation. As part of the study of supportive care, we will examine the implications of an introduction of patient-reported measures (PRMs) into care, adding surveys to interviews before and after PRMs roll-out. Data analysis will account for site-specific and cross-site issues using an adapted Qualitative Rapid Appraisal, Rigorous Analysis approach. Quantitative data from clinician surveys will be statistically analysed and triangulated with the related qualitative study findings.

Ethics and dissemination

For peer review only

Ethical approval was granted by South Eastern Sydney Local Health District Human Research Ethics Committee (no. 18/207). Findings will be shared with participating hospitals and widely disseminated through publications and presentations.

For peer review only

For peer review only - http://bmjopen.bmj.com/site/about/guidelines.xhtml

STRENGTHS AND LIMITATIONS OF THIS STUDY

- Multiple data types and sources are integrated to characterise MDC in different settings.
- A two-stage roll-out facilitates better targeting of observations and interviews in the second stage.
- A strategy of intensive data collection using multiple methods is used to ensure that site-specific data is interpreted within its local context.
- Findings will be based on a rigorous and extensive observational characterisation of Australian oncology outpatient services.

For peer review only

For peer review only - http://bmjopen.bmj.com/site/about/guidelines.xhtml

INTRODUCTION

In the developed world, invasive cancer is the leading cause of death.[1] Globally, the incidence of cancer is increasing;[2] in Australia, cancer incidence is projected to reach 150,000 new cases per year by 2020.[3] Survival rates are improving, with over 400,000 Australians living following a cancer diagnosis in the previous 5 years.[3] Cancer may be understood as a condition requiring medical and supportive care services from diagnosis through long-term survivorship.[4] Multidisciplinary care (MDC) is accepted as a best practice model of care provision for oncology services in Australia, and in advanced health systems globally.[5-9] MDC seeks to promote equitable, evidence-based care by a team that combines relevant expertise and enable patients to be involved in decision-making concerning their care.[6,10]

Work has been undertaken to translate MDC principles into practice; but significant challenges remain. Substantial evidence points to gaps between optimal evidence-based care and current care.[11-15] A review of research literature, national health sector reports and local cancer plans identified three key interconnected elements of MDC: diverse professional expertise; clinical decision-making; and, addressing patients' supportive care needs. In Figure 1 the coordination of care is positioned to reflect the way in which it binds and encompasses these elements.

<INSERT FIGURE 1. HERE>

Source: Authors' conceptualisation of MDC

Patient needs

In addition to medical treatment, people with cancer also need medical (e.g., pain relief) and non-medical supportive care (e.g., information needs). The challenges of addressing supportive needs are an ongoing concern in oncology.[16-18] Meeting these needs requires

For peer review only - http://bmjopen.bmj.com/site/about/guidelines.xhtml

identification, prioritisation, treatment planning and timely access to relevant professional expertise.[16-19] In general, identification of support needs in cancer care has not been systematic. Rather, routine care has relied on the clinical acumen of individual clinicians who identify needs during consultations and make appropriate referrals. Language spoken and level of health literacy also remain challenges in order to “pitch” information at the level most suitable for each patient. Routinely collecting information from patients, through Patient Reported Measures (PRMs), has been promoted as a mechanism to identify unmet supportive needs.[20-22] The Edmonton Symptom Assessment Scale[23], Distress Thermometer[24] are PRMs which will be introduced into routine cancer care in some of the outpatient clinics (OPCs) during the period of study, likely changing the management of patients’ supportive needs.[25-27] This study focuses on the provider perspective; the patient’s support networks are also important for addressing supportive needs, but these are beyond the study’s scope.

Diverse professional expertise

MDC is overseen by a core team which, while varying by cancer type, usually consists of pathologists, radiologists, medical oncologists, radiation oncologists, surgeons, nurses and allied health professionals.[6,10,28] Other specialist expertise can also be part of the core team, or can be added as required. This may include expertise such as in palliative care or pre- and rehabilitation over the course of the patient’s journey.[6] This approach is intended to utilise cross-disciplinary expertise to the fullest benefit of the patient and offer shared decision-making mechanisms for clinicians.[10,29] To develop an understanding of MDC we therefore need to harness the views of a diverse range of professionals and observe interprofessional collaboration in practice.

Decision-making

Multidisciplinary team meetings (MDTMs) are a fulcrum for the clinical coordination of MDC.[30,31] Research suggests that MDTM discussions influence diagnosis, staging and

For peer review only - http://bmjopen.bmj.com/site/about/guidelines.xhtml

treatment planning.[32-34] It is important to understand the organisation of MDTMs and the resources required to support them. Studies reveal diversity in the organisation of MDTMs, who is discussed and when, and the ways that clinicians engage to support clinical decision-making.[9] While MDTMs play a central role, clinicians make decisions outside MDTMs, without any consultation during routine care provision, or in consultation with other clinicians who may or may not be part of the team. In addition, decisions can be made at the meetings but evidence suggests that the recommendations are not always followed and in many meetings there can be disagreements in opinion.[35] How these issues are managed with the patient are likely to be highly individualised. We also seek to understand this behaviour.

Coordinated care

Among the top priorities for coordination are: 1) ongoing screening of patients' supportive needs and appropriate care in response and 2) the timely and appropriate delivery of care across the patient's journey.[36] Public hospital OPCs are tasked with providing care for diverse types of cancer across the patient journey, from diagnosis, through treatment and into survivorship. The models of coordination employed may be non-uniform; hybrid approaches may be used within a setting (e.g., simultaneously offering tumour stream-specialised care coordination, with generalised care coordinators covering all others).[37,38] Sub-optimal coordination can lead to fragmented care, conflicting information provided to patients, impaired access to appropriate services and inefficient use of the time and energy of health professionals and patients.[19,39,40]

Research framework

The investigation described in this paper is the first major phase of field research undertaken by the newly established National Health and Medical Research Council (NHMRC) Centre for Research Excellence in Implementation Science in Oncology (CRE-ISO), administered by the Australian Institute for Health Innovation at Macquarie University. In

For peer review only - http://bmjopen.bmj.com/site/about/guidelines.xhtml

healthcare, implementation science focuses on understanding processes and methods for supporting the uptake and integration of effective interventions to improve healthcare practices and outcomes.[41] The research described here will characterise MDC provision in two Sydney metropolitan Local Health Districts (LHDs), government agencies responsible for managing and providing public health services within a specified geography, usually of approximately one million residents in order to provide a foundational understanding of the realities of oncology service provision. This understanding will inform subsequent phases of research undertaken by the Centre and will guide the selection of implementation approaches, some of which will focus on developing and testing tailored interventions to address identified evidence-practice gaps. Health economics studies will assess the potential costs and benefits of alternative improvement effort options to inform implementation, and evaluate the costs and benefits associated where implemented.

Critical to this study is engagement with the context of service provision and its complexity; of the "interacting and interdependent" components of a health service that comprise MDC provision in hospitals.[42] Multiple sources of data will be harvested and integrated, exploring the formalised processes and local practices undertaken within the interconnected networks of semi-autonomous professionals that together shape care provision.[43] Systems of care provision in these settings are rarely static, and opportunities will be taken to examine the uptake of new practices, such as the introduction of PRMs, to learn about the processes that inhibit or promote effective adoption. This research will also seek to identify generic barriers and facilitators that impede or promote best practice, as background information to assist with the design of future interventions, and health economics studies.

Research Aims (RA)

The study aims to:

For peer review only - http://bmjopen.bmj.com/site/about/guidelines.xhtml

RA1. Develop understanding of cancer services in OPCs by characterising the organisation and practice of MDC;

RA2. Explore, in-depth, selected key elements of MDC:

i. Clinical decision-making in MDC and how doctors engage with decision-making support mechanisms in practice; and

ii. How health professionals engage with patients' supportive needs before and immediately after the introduction of PRMs.

RA3. Identify general barriers to, and facilitators of, the provision of MDC.

For peer review only

For peer review only - http://bmjopen.bmj.com/site/about/guidelines.xhtml

METHODS & ANALYSIS

A mixed-methods research design will be adopted: largely comprised of a variety of qualitative methods, complimented by a single quantitative survey. Methods adapted from ethnography will be harnessed, to facilitate intensive and efficient data collection; rapid ethnography will be used to reduce the burden of research on organisations and participants while maintaining rigour and richness.[44,45] Careful thought has been given to the demands of multi-site research and we adopt recommended strategies used to enhance validity and maintain capacity to adequately engage with the complexities of the settings.[46]

The methodological principles embedded in the design are: exploring everyday practices and the organisational relations that shape them, by observing participants at work in OPC settings; harnessing a plurality of participant perspectives, by interviewing professionals about their work; and generating rich descriptive accounts of MDC contexts by using analytical and interpretative approaches formulated for multi-site data.[47] A critical realist philosophical position is taken, which is underpinned by a realist ontological assumption and a critical epistemology.[48,49] From this position it is assumed that a given account may not perfectly reflect reality; rather, accounts may be contextually and structurally mediated, offering partial insight into complex phenomena.[50,51]

Design

Implementation will be staged. Prior to study commencement, profiles and structural descriptions of each hospital setting were developed, using publicly-available information. In *Stage One,* an overview of the organisation of practice and models of care will be derived through interviews with "Navigators" (e.g., OPC staff with an overview of care pathways such as Nurse Unit Managers or care coordinators), review of unpublished policies and procedures and unstructured observations of care provision. In *Stage Two*, we enrich the accounts of these

For peer review only - http://bmjopen.bmj.com/site/about/guidelines.xhtml

settings through additional observation in OPCs. Together, these methods address the first research aim (RA1) and provide a foundation for the second (RA2) and third (RA3).

Stage Two also includes focused studies, undertaken in parallel. In Study 2.1, interviews with doctors and observations of cancer MDTMs provide insight into clinical decision-making practices in MDC and how doctors engage with decision-making support mechanisms (RA2.i). Engagement with patients' supportive needs will be explored in Study 2.2 (RA2.ii), using a two part, mixed-method design consisting of interviews with health professionals, observation of cancer MDTMs and a "clinician readiness" survey; all research elements will be performed before introduction of new PRMs, and repeated after they have been introduced.

Barriers and facilitators to the provision of MDC (RA3) will be elicited as part of the interviews in each study and enhanced by our observational studies in *Stages One* and *Two*. The study design is informed by the Consolidated Criteria for Reporting Qualitative Research.[52] The study will run from the 28th of February, 2019 up to the 31st of December 2020.

Selection of sites and participants

The investigation will be undertaken within two metropolitan LHDs, each containing three participating public hospitals with OPCs. Collectively, the OPCs offer an extensive range of consultation, coordination, wellness and outpatient treatment services. Each LHD contains two large hospitals which provide an extensive range of services and a smaller hospital offering a more limited range of services, closely linked with larger centres (e.g., including the provision of teleconferencing for meetings).

Sites and meetings: In *Stage One* all sites will be studied, to understand each hospital setting. In *Stage Two*, maximum variation sampling[53] will be used to allocate the 'continuing observation' sessions to participating sites. As there are differences in the services between sites (e.g. some hospitals offer a smaller range), observation sessions will be allocated to best

For peer review only - http://bmjopen.bmj.com/site/about/guidelines.xhtml

capture the diverse range of practices and processes useful in the characterisation of MDC within and across sites. Purposive sampling will be used to select the MDTMs approached to participate. We will generate a sample comprising a range of tumour streams including common and rarer cancers. Study 2.1 will largely be conducted in one LHD, with study 2.2 in the other.

Participants: The participant inclusion criteria for interviews, surveys and health professional observations is generally broad: core medical staff (e.g., medical oncologists, surgeons, radiation oncologists); nursing staff (e.g., clinical nurse consultants, cancer care coordinators); and allied health staff (e.g., social workers, psychologists). However, participation in *Stage One* "Navigator interviews" will be restricted to staff with an overview of care pathways (e.g., cancer care coordinators, Nurse Unit Managers, managers of clinical streams). Similarly, in Study 2.2 the "clinician decision-making" interviews will be undertaken with specialist doctors (e.g. medical oncologists, surgeons, radiation oncologists) working in cancer services.

Recruitment: Information about the research including its aims, an introduction to the researchers undertaking data collection, contact details and participant information sheets will be disseminated through email, flyers and posters, and through information sessions for target professional groups at each site. Snowball sampling will also be used; participants will be encouraged to discuss the project with colleagues. Letters will be sent to the Chairpersons of cancer MDTMs and to the authorities responsible for the OPCs, to provide information about the research and request permission for the observations. These letters will include "Health Professional Information Sheets" which recipients will be encouraged to share with relevant colleagues as a component of pre-approval consultation.

Sample size ranges: The Study design features, presented in Table 1, summarise the stages of the research, the data collected in each stage and the target number of observations,

For peer review only - http://bmjopen.bmj.com/site/about/guidelines.xhtml

interviews and surveys. The sample sizes designated for the interview components are guided by principles identified in reported research literature, as well as Malterud, Siersma and Guassora's (2016) model for generating "information power" in qualitative studies. The model accounts for the following factors when estimating a sample range: the study aim; specificity of the sample; the envisioned quality of the interview interaction; analysis strategy; and established theory.[54] The number of proposed interviews in Study 2.2. is also guided by the sample size needed for the clinician readiness survey that is undertaken as part of the interview. The sample size allocated for the individual health professional observations reflects the narrow and specific focus of gaining a deep understanding of the roles these professionals play in MDC. The number of sessions proposed for the observations of the OPCs and MDTMs are comparable with similar qualitative studies[55] and are appropriate for multi-site ethnographic studies.[56]

Research Team: The investigation will be overseen by a team with extensive experience in health services and quality improvement research, including leaders in implementation science and oncology. Fieldwork will be guided by principal investigators with direct clinical and research experience in oncology service provision. Data will be collected by GA (Senior Research Fellow, Ph.D., male), BNGE (Research Fellow, Ph.D., female) and TW (Research Associate, Masters, female) who are trained in qualitative, quantitative and mixed-methods investigation. Each of these researchers has fieldwork experience across a range of health services. Qualitative analysis will be conducted by BNGE and TW, while YT (Research Fellow, Ph.D., female) will analyse quantitative data. Appropriately qualified health professionals who join the research team for *Stage Two* will be trained and supervised.

Table 1. Study design features

For peer review only - http://bmjopen.bmj.com/site/about/guidelines.xhtml

For peer review only

Phase/Stage	Text	Interviews	Survey	MDTM Observations	Undirected Observation Sessions*
**Stage One**					
**Stage One Multi-site characterisation**	Review of policies and procedures relevant to interview discussion	• Navigator (n=8-16)<br>• Decision-making (n=3-8)<br>• Supportive needs (n=4-10)	n/a	Cancer MDTMs (n=3-8)	Initial:<br>• OPCs (n=15-25)<br>• Individual (n=3-6)
**Stage Two**					
**Study 2.1 Decision-making**	Review of policies and procedures relevant to interview discussion	Decision-making (n=15-30)	n/a	Cancer MDTMs (n=3-8)	Ongoing:<br>• OPCs (n=15)<br>• Individual (n=7)
**Study 2.2. Supportive needs**		• Baseline (n=15-30)<br>• Follow-up (n=15-30)	• Baseline (n=30-50)<br>• Follow-up (n=30-50)	Cancer MDTMs (n=3-8)	
**Estimated totals**	n/a	60- 124	30-50	9-24	• OPCs (n=30-40)<br>• Individual (n=10)

Table key	
	Undertaken at all sites
	Predominantly undertaken in LHD 1
	Predominantly undertaken in LHD 2
*	1 observation session= half day or 3-4hrs

Legend: MDTM=Multidisciplinary Team Meeting; OPC=cancer outpatient clinic.

* 1 observation session= half day or 3-4hrs

Data Collection

As part of protocol development, reviews of the literature were conducted to conceptualise and identify key issues in MDC provision and to scope the methodological scholarship relevant to the design. Publicly-available annual reports and cancer plans from each health district were analysed to generate a description of cancer services at each hospital. These profiles were used to map key areas for data collection. Implementation science approaches emphasise the importance of the local context of adoption and engagement with its stakeholders.[41-43] During this stage, input was sought from oncology management to ensure the local relevance of the research and the feasibility of the proposed methods.

For peer review only

Stage One. Multi-site characterisation study

Interviews: Semi-structured "Navigator" interviews will be undertaken with professionals who have overview roles (e.g., cancer care coordinator), oriented by care pathway maps, to establish the models of care and processes involved in the patient journey (n=8-16).[57,58] The care pathway maps were derived from the stages of MDC depicted by Fennell, Das, Clauser, Petrelli and Salner (2010),[59] focused on the diagnosis and OPC treatment stages (Figure 2). Through the interviews, maps will be reworked to reflect the Australian services context, and the differing trajectories followed by different: a) tumour streams (accounting for tumour-specific services); and b) risk categories and complexity. Where interviewees refer to specific policies and procedures, documentation will be formally requested to help situate the discussion within its policy context. Interviews are expected to take approximately 40 minutes and will be audio-recorded and transcribed.

Observations: Three types of observations will be made in the research:

- Unstructured observations of OPCs will provide an understanding of the real-world, routine organisation and actual delivery of MDC in these settings (e.g., we will observe *ad hoc* and planned collaboration between professionals from differing disciplines during the care delivery process; n=15-25).

- Observations of individual health professionals will capture the key activities undertaken, to understand how their work contributes to the provision of MDC (n=3-6 individual health professionals observed for half or all of their day).

- Observations of MDTMs will be conducted using a semi-structured template (Figure 3) informed by previous MDTM observational research to guide what we observe and record, and systematise our description of meetings (n=3-8 meetings).[30,60] Fieldnotes will record observations of: professional groups in attendance; specific roles; support provided to

For peer review only - http://bmjopen.bmj.com/site/about/guidelines.xhtml

administer the meeting (i.e. technology); organisation of cases; clinical decision-making; patient-centred care, and; organisation of follow-up.

All observations will be recorded in hand-written fieldnotes which will be used to generate rich accounts of care.

<INSERT FIGURE 2. HERE>

Source: Fennell et al. (2010)[59] (adapted from Zapka et al., 2003)[61]

Transition to Stage Two: During *Stage One*, we will pilot elements of the clinician interviews about decision-making (n=3-8) and patients' supportive needs (n=4-10) to be undertaken in *Stage Two,* and interview prompts will be refined. Where eligible health professionals indicate interest in joining the research team for *Stage Two* fieldwork, they will be briefed and provided with necessary training and support. Modifications to methods and the research team composition will be submitted for ethical approval, if required.

<INSERT FIGURE 3: HERE>

Source: Developed by authors based on Harris et al (2016)[60] and Rankin et al. (2018)[30]

Stage Two. MDC Studies

This stage will enrich accounts of MDC provision via "ongoing characterisation" (i.e. n=3-6 further observations of individual health and n=15 unstructured observations of OPCs), at the same time contextualising the focused studies detailed below.

For peer review only - http://bmjopen.bmj.com/site/about/guidelines.xhtml

Study 2.1 Clinical decision-making

Interviews: Doctors will participate in semi-structured interviews that explore their decision-making practices and their engagement with support mechanisms (n=15-30). Topics will include their perceptions of decision-making mechanisms, the decision-making support available to them, their own decision-making practices and their use of MDTMs at key points in the patient journey. A conceptual map of the patient journey (Figure 4) will be used to orient the interview, with questions tailored to each stage. Barriers to and drivers of efficient clinical decision-making will be explored. Interviews are expected to take approximately 40 minutes and will be audio-recorded and transcribed.

<INSERT FIGURE 4. HERE>

Source: Authors' adaption of Fennell et al. (2010)[59]

Observation of MDTMs: Meetings will be observed using a semi-structured template (Figure 3), focussing on the "clinical decision-making" component (n=3-8). Observational data will capture information about this decision-making mechanism and contextualise interviews.

Study 2.2 Patients' supportive needs

Interviews: Prior to introduction of PRMs, semi-structured interviews will be conducted with health professionals about how they engage with patients' supportive needs in their current practice (n=15-30). Topics will include: models of care; their individual role; identification and screening; management; and any barriers and facilitators experienced in identifying and responding to supportive needs. The interviews will also explore preparations for and expectations of PRMs.

For peer review only - http://bmjopen.bmj.com/site/about/guidelines.xhtml

Following the adoption of PRMs, health professionals will again participate in semi-structured interviews (n=15-30). These will focus on the adoption and implications of PRMs in relation to the topics addressed in pre-introduction interviews. Pre- and post-introduction interviews are expected to take approximately 40 minutes and will be audio-recorded and transcribed.

Surveys: An eight-item clinician readiness survey (Willis et al. 2009)[62] will be completed by health professionals before and after the adoption of PRMs (n=30-50 in each phase). The survey will assess the current level of preparedness, and changes over time. This survey will take approximately five minutes to complete and will be performed as part of the interviews and will also be distributed more widely to achieve the desired sample size.

Observation of MDTMs: Meetings will be observed using a semi-structured template (Figure 3), focussing on the "patient-centred care & supportive care needs" component (n=3-8). Data will capture information about engagement with supportive needs in MDTMs and contextualise the interviews.

Data Analysis

Interview data: Basic descriptive data collected during interviews (e.g. listing of team members involved in different points of the patient journey) will be collated across interviews then categorised and organised within a matrix. The discursive data will be coded by two researchers and thematically analysed, oriented by an inductive approach.[63] Where process maps are used (e.g. navigator and decision-making interviews), these will frame the analysis. Constant comparative analysis will be applied to interpret data gathered before and after the introduction of PRMs. This strategy accounts for the cross-site, multiple-source nature of the data. Our approach for making sense of cross-site data is informed by the "Qualitative Rapid Appraisal, Rigorous Analysis" methodology developed by Phillips et al. (2014).[45] First, all

For peer review only - http://bmjopen.bmj.com/site/about/guidelines.xhtml

"like" data (e.g., all interview data) is analysed at a site, followed by intra-site analysis of all data from one OPC. Inter-site analysis is then employed to consider the data across the sites.[45]

Observational data: Fieldnotes will be indexed to identify the specific setting/MDTM meeting, key processes and events and study focus. The notes will be integrated within hospital cancer service profiles to elaborate descriptions of OPC settings (i.e., the organisational features of care). Fieldnotes from MDTM and OPC observations will then be developed into accounts that contextualise data collected in the focused studies. Data collected from individual health professional observations will be developed into an account of the role.

Clinician readiness survey data: Analysis will include a repeated-measures ANOVA to test for differences in clinician attitudes before and after PRMS roll-out. If possible, subgroup analyses will test for differences in attitudes between different demographic and clinical subgroups. The quantitative data will be used to complement the interview data on this topic. Based on known effect sizes from results of the Willis et al. (2009)[62] paper using the Outcome Measurement Questionnaire to assess change in response to training, using a power of 0.8 and alpha of 0.05, the minimum sample size was estimated as n=32 participants (i.e., to detect a change in mean scale score from 4.09 pre- to 4.49 post-introduction, with a standard deviation of 0.57).

Measurement of outcomes

The main outcomes of the investigation are derived from the aims. Through this investigation we will generate a rich and comprehensive description of the organisation and practice of MDC. We will document practices of clinical decision-making including the role of the MDT in such practice. We will produce an account of how patient supportive needs are engaged with and the implications that the introduction of PRMs has for this engagement. The factors which impede or promote practice will be identified and used to inform subsequent CRE-ISO activity.

For peer review only - http://bmjopen.bmj.com/site/about/guidelines.xhtml

For the qualitatively-driven research, assessment of the outcomes involves judgement of the adequacy with which the research aims were addressed. This is largely reliant on the quality of the data collected and the rigour of its analysis. Sufficiently detailed data are needed from a range of sources to produce rich descriptions of MDC practice, including its diversity. Fieldwork and analytic strategies should allow findings to be situated within OPC contexts as well as revealing points of connection and contradiction between sites. Characterisation of MDC provision will be achieved through credible, adequately complex and well-grounded accounts. Multi-method data collection (including a quantitative component) allows for consideration of various sources of input, and triangulation; a team-based approach to analysis will facilitate ongoing reflection and monitoring.

For peer review only

For peer review only - http://bmjopen.bmj.com/site/about/guidelines.xhtml

ETHICS & DISSEMINATION

Ethical approval was granted by South Eastern Sydney LHD Human Research Ethics Committee (#18-207). Informed written consent will be obtained from interviewees and from health professionals who are individually observed. Interview and observation arrangements will be made with health professionals at least one week in advance to ensure they have time to prepare. Participants may withdraw, without justification, up to one-week post interview/observation or whenever the data is de-identified.

Posters will be displayed, and information sheets will be available during observational sessions so that those present can raise concerns or decline participation. This can be done at any time up until the observational data is collected. The research team will not collect any information about patients and, guided by health professionals, will minimise exposure to direct patient interactions. Researchers will answer questions posed about their presence and provide information sheets that contain ethics committee details. In the event of participants experiencing discomfort or distress arising from the research, researchers will facilitate access to appropriate support services.

Data storage and protection: Data will be stored on a password-protected computer and on a secure University server, accessible only to the research team. Data will be de-identified and separated from participant identifiers. Research records will be retained for at least five years post-study completion or last publication, in accordance with Section 2.1.1 of the Australian Code for the Conduct of Responsible Research (2007)[64] before secure destruction.

Dissemination: Findings will be disseminated to academics, professionals and the public through publications and presentations. Tailored reports will be provided to participating hospitals and presentations given to participating professional groups.

For peer review only - http://bmjopen.bmj.com/site/about/guidelines.xhtml

Patient and Public Involvement Statement

The coordinating research institution routinely consults with patients, their representatives and the general public, to ensure that adequate input is secured for research programs; a key partner is the Consumers Health Forum of Australia. This specific project was developed without detailed patient involvement; patients were not invited to comment on the study design or to the writing or editing of this protocol. This project will help us to describe service structure and organisation, and to learn from the perspectives of MDC providers. The experiences of patients are vital to understanding these services, including their views on the barriers and facilitators to high-quality care. Patient experiences will be engaged with in dedicated, patient-centred studies within the CRE-ISO program of research. For example, in preparation, we have already looked at data from UK Patient Experience Surveys[65], and are in the process of accessing Australian data from a similar source.

For peer review only

For peer review only - http://bmjopen.bmj.com/site/about/guidelines.xhtml

IMPACT & SIGNIFICANCE

The research seeks to capture a diverse range of practices and processes useful in the characterisation of MDC, with attention to site-specific nuances that may be relevant to future improvement efforts or health economic studies. The accumulative characterisation will be combined with focused, in-depth studies of clinical decision-making and processes for meeting patients' supportive care needs. The research involves rigorous and extensive observations of Australian public oncology OPCs and MDTMs; it offers a substantial opportunity for the generation of new and critical insights. The findings will serve as a foundation for the design of studies to address identified evidence-practice gaps in cancer care.

For peer review only

References

1. Jemal A, Bray F, Center MM, et al. Global cancer statistics. *CA Cancer J Clin* 2011;61:69-90. doi:10.3322/caac.20107

2. International Agency for Research on Cancer. GLOBOCAN 2018 Global Cancer Observatory, 2018. https://gco.iarc.fr/ (accessed 4 July 2019).

3. Australian Institute of Health and Welfare. Cancer incidence projections, Australia 2011 to 2020. Canberra, Australia: AIHW, 2012.

4. Australian Institute of Health and Welfare. Cancer in Australia. Canberra, Australia: AIHW, 2017.

5. American College of Surgeons: Commission on Cancer. Cancer program standards: ensuring patient-centered care. Chicago, IL: American College of Surgeons, 2016.

6. Cancer Australia. All about multidisciplinary care. https://canceraustralia.gov.au/clinical-best-practice/multidisciplinary-care/all-about-multidisciplinary-care (accessed 20 July 2018).

7. Jiang H. Patients should surely be included in multidisciplinary team meetings in China healthcare. *BMJ* [eLetter] 14 October 2015. https://www.bmj.com/content/351/bmj.h5295/rr-1

8. Pan C-C, Kung P-T, Wang Y-H, et al. Effects of multidisciplinary team care on the survival of patients with different stages of non-small cell lung cancer: a national cohort study. *PloS One* 2015;10:e0126547-e47. doi:10.1371/journal.pone.0126547

9. Saini KS, Taylor C, Ramirez AJ, et al. Role of the multidisciplinary team in breast cancer management: results from a large international survey involving 39 countries. *Ann Oncol* 2012;23:853-59. doi:10.1093/annonc/mdr352

For peer review only

For peer review only - http://bmjopen.bmj.com/site/about/guidelines.xhtml

10. Zorbas H, Barraclough B, Rainbird K, et al. Multidisciplinary care for women with early breast cancer in the Australian context: what does it mean? *Med J Aust* 2003;179:528-31.

11. Barton MB, Jacob S, Shafiq J, et al. Estimating the demand for radiotherapy from the evidence: a review of changes from 2003 to 2012. *Radiol Oncol* 2014;112:140-44. doi:10.1016/j.radonc.2014.03.024

12. Batumalai V, Shafiq J, Gabriel G, et al. Impact of radiotherapy underutilisation measured by survival shortfall, years of potential life lost and disability-adjusted life years lost in New South Wales, Australia. *Radiol Oncol* 2018;129:191-95. doi:10.1016/j.radonc.2018.06.026

13. Delaney GP, Barton MB. Evidence-based estimates of the demand for radiotherapy. *Clin Oncol* 2015;27:70-76. doi:10.1016/j.clon.2014.10.005

14. Hanna TP, Shafiq J, Delaney GP, et al. The population benefit of evidence-based radiotherapy: 5-Year local control and overall survival benefits. *Radiol Oncol* 2018;126:191-97. doi:10.1016/j.radonc.2017.11.004

15. Telli ML, Jensen KC, Vinayak S, et al. Phase II study of gemcitabine, carboplatin, and iniparib as neoadjuvant therapy for triple-negative and BRCA1/2 mutation–associated breast cancer with assessment of a tumor-based measure of genomic instability: PrECOG 0105. *J Clin Oncol* 2015;33:1895-901. doi:10.1200/jco.2014.57.0085

16. Dilworth S, Higgins I, Parker V, et al. Patient and health professional's perceived barriers to the delivery of psychosocial care to adults with cancer: a systematic review. *Psychooncology* 2014;23:601-12. doi:10.1002/pon.3474

17. Rankin NM, Barron JA, Lane LG, et al. Psychosocial oncology services in New South Wales. *Aust Health Rev* 2011;35:156-63. doi:10.1071/AH08730

For peer review only - http://bmjopen.bmj.com/site/about/guidelines.xhtml

18. Walsh J, Young JM, Harrison JD, et al. What is important in cancer care coordination? a qualitative investigation. *Eur J Cancer Care* 2011;20:220-27. doi:10.1111/j.1365-2354.2010.01187.x

19. Walsh J, Harrison JD, Young JM, et al. What are the current barriers to effective cancer care coordination? a qualitative study. *BMC Health Serv Res* 2010;10:132. doi:10.1186/1472-6963-10-132

20. Basch E, Deal AM, Kris MG, et al. Symptom monitoring with patient-reported outcomes during routine cancer treatment: a randomized controlled trial. *J Clin Oncol* 2016;34:557-65. doi:10.1200/jco.2015.63.0830

21. Luckett T, Butow PN, King MT. Improving patient outcomes through the routine use of patient-reported data in cancer clinics: future directions. *Psychooncology* 2009;18:1129-38.doi:10.1002/pon.1545

22. Williams K, Sansoni J, Morris D, et al. Patient-reported outcome measures: literature review. Wollongong, Australia, Australian Health Services Research Institute, University of Wollongong, 2016.

23. Watanabe SM, Nekolaichuk CL, Beaumont C. The Edmonton Symptom Assessment System, a proposed tool for distress screening in cancer patients: development and refinement. *Psychooncology* 2012;21:977-85. doi:10.1002/pon.1996

24. National Comprehensive Cancer Network (NCCN). NCCN Clinical Practice Guidelines in Oncology (NCCN Guidelines®) for distress management 2016.

25. Cancer Institute NSW. Patient-reported measures (PRMs) 2018. https://www.cancer.nsw.gov.au/how-we-help/quality-improvement/patient-reported-measures (accessed 30 August 2018).

26. Girgis A, Delaney GP, Miller AA. Utilising ehealth to support survivorship care. *Cancer Forum* 2015; 39:86-89.

For peer review only - http://bmjopen.bmj.com/site/about/guidelines.xhtml

27. Girgis A, Durcinoska I, Levesque JV, et al. eHealth system for collecting and utilizing patient reported outcome measures for personalized treatment and care (PROMPT-Care) among cancer patients: mixed methods approach to evaluate feasibility and acceptability. *J Med Internet Res* 2017;19:e330. doi:10.2196/jmir.8360

28. Lamprell K, Arnolda G, Delaney GP, et al. The challenge of putting principles into practice: resource tensions and real-world constraints in multidisciplinary oncology team meetings. *Asia Pac J Clin Oncol* Published Online First: 21 May 2019. doi:10.1111/ajco.13166

29. National Breast Cancer Centre. Multidisciplinary care (MDC) in Australia: a national demonstration project in breast cancer. Camperdown, NSW, National Breast Cancer Centre, 2003.

30. Rankin NM, Lai M, Miller D, et al. Cancer multidisciplinary team meetings in practice: results from a multi-institutional quantitative survey and implications for policy change. *Asia Pac J Clin Oncol* 2018;14:74-83. doi:10.1111/ajco.12765

31. Wilcoxon H, Luxford K, Saunders C, et al. Multidisciplinary cancer care in Australia: a national audit highlights gaps in care and medico-legal risk for clinicians. *Asia Pac J Clin Oncol* 2011;7:34-40. doi:10.1111/j.1743-7563.2010.01369.x

32. Brunner M, Gore SM, Read RL, et al. Head and neck multidisciplinary team meetings: effect on patient management. *Head Neck* 2015;37:1046-50. doi:10.1002/hed.23709

33. Davies AR, Deans DAC, Penman I, et al. The multidisciplinary team meeting improves staging accuracy and treatment selection for gastro-esophageal cancer. *Dis Esophagus* 2006;19:496-503. doi:10.1111/j.1442-2050.2006.00629.x

34. Friedland PL, Bozic B, Dewar J, et al. Impact of multidisciplinary team management in head and neck cancer patients. *Br J Cancer* 2011;104:1246–1248. doi:10.1038/bjc.2011.92

For peer review only - http://bmjopen.bmj.com/site/about/guidelines.xhtml

35. Hamilton DW, Heaven B, Thomson RG, et al. Multidisciplinary team decision-making in cancer and the absent patient: a qualitative study. *BMJ Open* 2016;6:e012559. doi:10.1136/bmjopen-2016-01255934.

36. Shaw T, York S, White K, et al. Defining success factors to describe coordinated care in cancer. *Transl Behav Med* 2018;8:357-65. doi:10.1093/tbm/iby022

37. Eicher MRE, Marquard S, Aebi S. A nurse is a nurse? a systematic review of the effectiveness of specialised nursing in breast cancer. *Eur J Cancer* 2006;42:3117-26. doi:10.1016/j.ejca.2006.07.007

38. Porter-Steele J, Tjondronegoro D, Seib C, et al. 'Not one size fits all': a brief review of models of care for women with breast cancer in Australia. *Cancer Forum* 2017;41:13-19.

39. Kripalani S, Jackson AT, Schnipper JL, et al. Transitions of care at hospital discharge. *J Hosp Med* 2007;5:314-23. doi:10.1002/jhm.228

40. Weaver SJ, Jacobsen PB. Cancer care coordination: opportunities for healthcare delivery research. *Transl Behav Med* 2018;8:503-08. doi:10.1093/tbm/ibx07939.

41. Rapport F, Clay-Williams R, Churruca K, et al. The struggle of translating science into action: foundational concepts of implementation science. *J Eval Clin Pract* 2018;24:117-26. doi:10.1111/jep.12741

42. Braithwaite J, Marks D, Taylor N. Harnessing implementation science to improve care quality and patient safety: a systematic review of targeted literature. *Int J Qual Health Care* 2014;26:321-29. doi:10.1093/intqhc/mzu047

43. Braithwaite J, Churruca K, Long JC, et al. When complexity science meets implementation science: a theoretical and empirical analysis of systems change. *BMC Med* 2018;16:63. doi:10.1186/s12916-018-1057-z

44. Handwerker W. Quick Ethnography. Walnut Creek, CA: Altamira Press 2001.

For peer review only - http://bmjopen.bmj.com/site/about/guidelines.xhtml

45. Phillips CB, Dwan K, Hepworth J, et al. Using qualitative mixed methods to study small health care organizations while maximising trustworthiness and authenticity. *BMC Health Serv Res* 2014;14:559. doi:10.1186/s12913-014-0559-4

46. Millen D. Rapid ethnography: time deepening strategies for HCI field research. Proceedings of the 3rd Conference on Designing Interactive Systems: Processes, Practices, Methods, and Techniques; 2001 August 17-19; New York, USA: ACM, 2000. 45.

47. Charlesworth S, Baines D. Understanding the negotiation of paid and unpaid care work in community services in cross-national perspective: the contribution of a rapid ethnographic approach. *J Fam Stud* 2015;21:7-21. doi:10.1080/13229400.2015.1010263

48. Harper D. Choosing a qualitative research method. In: Harper D, Thompson AR, eds. Qualitative Research Methods in Mental Health and Psychotherapy: A Guide for Students and Practitioners. Hoboken, NJ: Wiley-Blackwell 2011: 83–98.

49. Bentall DPR. The medicalisation of misery: a critical realist analysis of the concept of depression. *J Ment Health* 1999;8:261-74. doi:10.1080/09638239917427

50. Harper D, Thompson AR. Qualitative Research Methods in Mental Health and Psychotherapy: A Guide for Students and Practitioners. Hoboken, NJ: Wiley-Blackwell 2011.

51. Willig C. Introducing Qualitative Research in Psychology. Maidenhead, UK: McGraw-Hill Education 2013.

52. Tong A, Sainsbury P, Craig J. Consolidated criteria for reporting qualitative research (COREQ): a 32-item checklist for interviews and focus groups. *Int J Qual Health Care* 2007;19:349-57. doi:10.1093/intqhc/mzm042

For peer review only - http://bmjopen.bmj.com/site/about/guidelines.xhtml

53. Palinkas LA, Horwitz SM, Green CA, et al. Purposeful sampling for qualitative data collection and analysis in mixed method implementation research. *Adm Policy Ment Health* 2015;42:533-44. doi:10.1007/s10488-013-0528-y

54. Malterud K, Siersma VD, Guassora AD. Sample size in qualitative interview studies: guided by information power. *Qual Health Res* 2015;26:1753-60. doi:10.1177/1049732315617444

55. Bridges J, Hughes J, Farrington N, et al. Cancer treatment decision-making processes for older patients with complex needs: a qualitative study. *BMJ Open* 2015;5:e009674. doi:10.1136/bmjopen-2015-009674

56. Renedo A, Marston C. Developing patient-centred care: an ethnographic study of patient perceptions and influence on quality improvement. *BMC Health Serv Res* 2015;15:122. doi:10.1186/s12913-015-0770-y55.

57. Lichtner V, Dowding D, Allcock N, et al. The assessment and management of pain in patients with dementia in hospital settings: a multi-case exploratory study from a decision making perspective. *BMC Health Serv Res* 2016;16:427. doi:10.1186/s12913-016-1690-1

58. Trebble TM, Hansi N, Hydes T, et al. Process mapping the patient journey: an introduction. *BMJ* 2010;341 doi:10.1136/bmj.c4078

59. Fennell ML, Prabhu Das I, Clauser S, et al. The organization of multidisciplinary care teams: modeling internal and external influences on cancer care quality. JNCI Monographs 2010;40:72-80. doi:10.1093/jncimonographs/lgq010

60. Harris J, Taylor C, Sevdalis N, et al. Development and testing of the cancer multidisciplinary team meeting observational tool (MDT-MOT). *Int J Qual Health Care* 2016;28:332-38. doi:10.1093/intqhc/mzw030

For peer review only - http://bmjopen.bmj.com/site/about/guidelines.xhtml

61. Zapka JG, Taplin SH, Solberg LI, et al. A framework for improving the quality of cancer care: the case of breast and cervical cancer screening. *Cancer Epidemiol Biomark Prev* 2003;12:4-13. 60.

62. Willis A, Deane FP, Coombs T. Improving clinicians' attitudes toward providing feedback on routine outcome assessments. *Int J Ment Health Nurs* 2009;18:211-15. doi:10.1111/j.1447-0349.2009.00596.x

63. Joffe H. Thematic analysis. In: Harper D, Thompson AR, eds. Qualitative Research Methods in Mental Health and Psychotherapy: A Guide for Students and Practitioners. Hoboken, NJ: Wiley-Blackwell 2011:209-223.

64. National Health Medical Research Council. Australian Code for the Responsible Conduct of Research: National Health and Medical Research Council, the Australian Research Council and Universities, Australia, 2007.

65. Tran Y, Lamprell K, Nic Giolla Easpaig B, et al. What information do patients want across their cancer journeys? a network analysis of cancer patients' information needs. *Cancer Med* 2019;8:155-64. doi:10.1002/cam4.1915

For peer review only - http://bmjopen.bmj.com/site/about/guidelines.xhtml

Declarations

Authors' contributions:

JB, GD, WL, RW, DC, IO, JK and JW led the conceptualisation of the work and the development of the design was led by JB, GD, WL, GA, BNGE, YT, MB, KL, RC and TW. With guidance from the other authors, BNGE wrote the first draft and all authors contributed to revising key content to develop the final version. All authors have seen and approve of the final manuscript.

Funding statement:

JB receives funding to support this work from NHMRC grants APP9100002 and APP1135048.

Competing interests statement

None to declare

For peer review only

List of tables and figures

Figure 1. Key elements of MDC

Figure 2. MDC interventions at transitions in cancer care

Figure 3. MDTM data collection template

Figure 4. Mapping of points of diagnosis and treatment decision-making in MDC

For peer review only

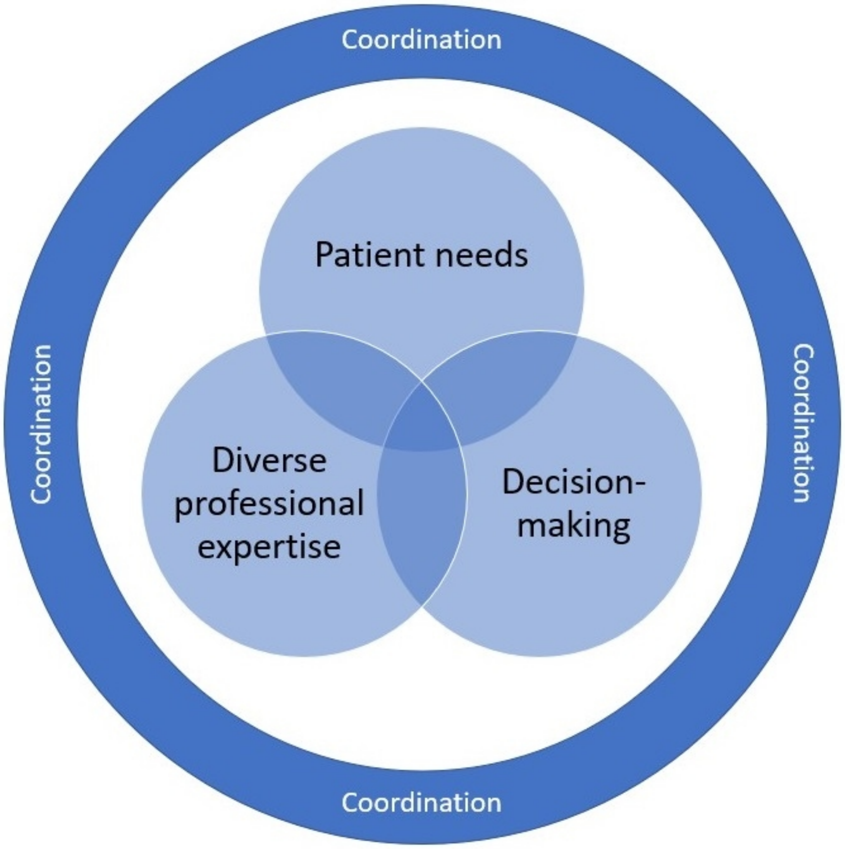

Source: Authors' conceptualisation of MDC

66x58mm (300 x 300 DPI)

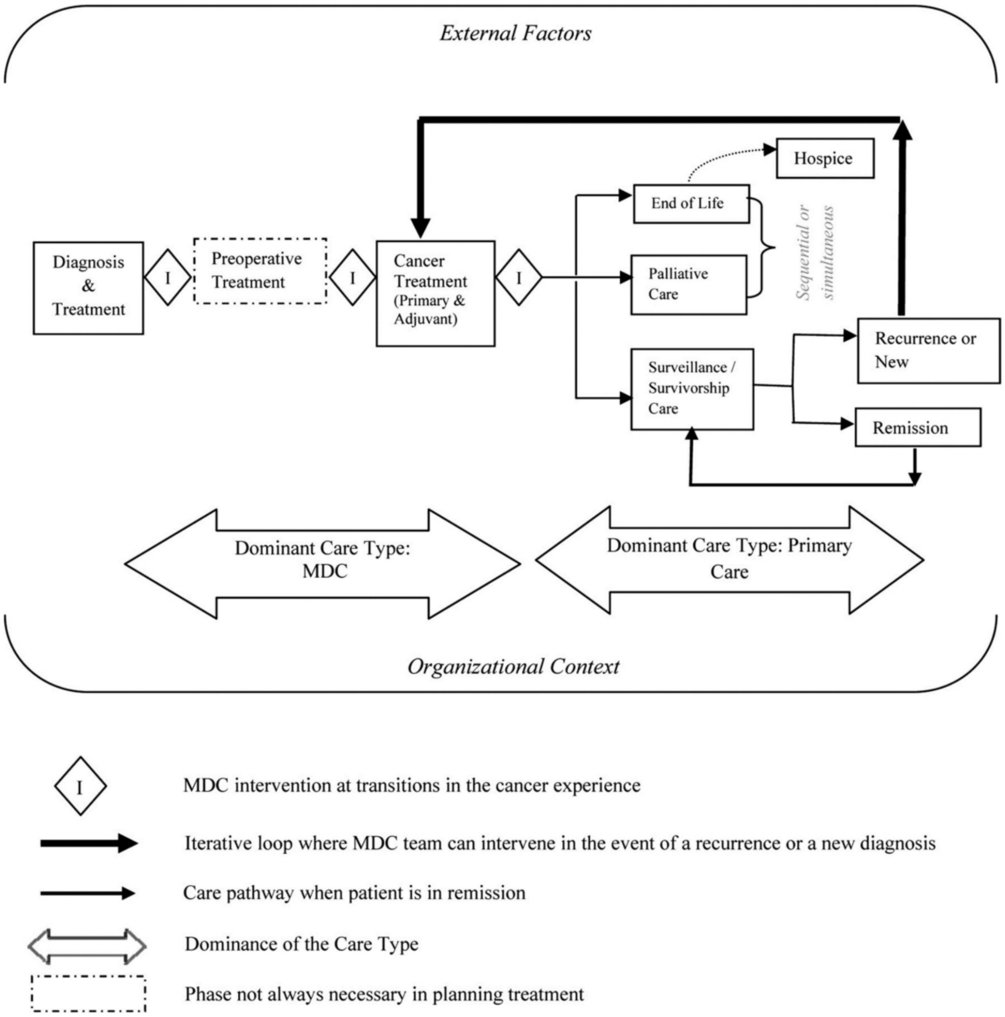

Source: Fennell et al. (2010)55 (adapted from Zapka et al., 2003)57

188x190mm (300 x 300 DPI)

MDTM & Hospital:<br>Date:	Notes
Attendance of health professional groups represented at MDT meetings by various health professional groups	
Chairperson and any meeting specific roles allocated	
Technological, equipment and administrative support in MDT meetings	
Organisation of the presentation of cases including<br>• The order<br>• Any prioritisation strategies<br>• Any patient inclusion criteria for meetings	
Clinical decision-making<br>• Discipline of professional input<br>• Types of information considered<br>• Evidence and/ or research incorporated into meetings	
Patient-centred care & Supportive care needs	
Organisation of any follow up tasks	

Source: Developed by authors based on Harris et al (2016)56 and Rankin et al. (2017)27

108x60mm (300 x 300 DPI)

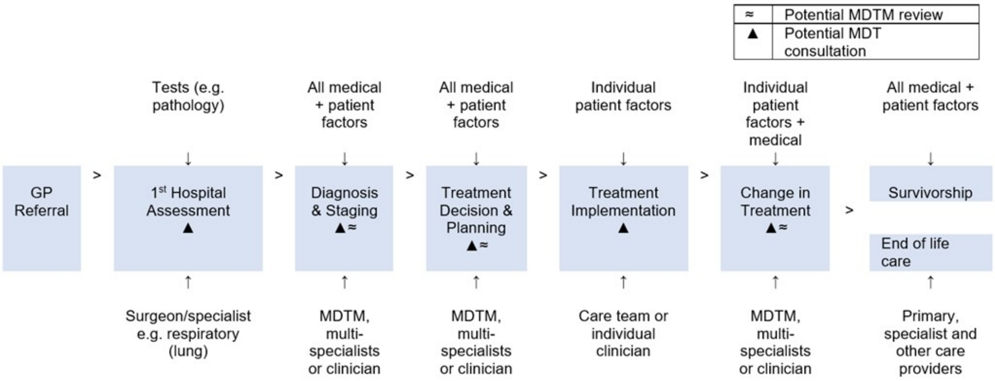

Source: Authors’ adaption of Fennell et al. (2010)55

86x33mm (300 x 300 DPI)

COREQ (COnsolidated criteria for REporting Qualitative research) Checklist

A checklist of items that should be included in reports of qualitative research. You must report the page number in your manuscript where you consider each of the items listed in this checklist. If you have not included this information, either revise your manuscript accordingly before submitting or note N/A.

Topic	Item No.	Guide Questions/Description	Reported on Page No.
**Domain 1: Research team and reflexivity**			
Personal characteristics			
Interviewer/facilitator	1	Which author/s conducted the interview or focus group?	
Credentials	2	What were the researcher's credentials? E.g. PhD, MD	
Occupation	3	What was their occupation at the time of the study?	
Gender	4	Was the researcher male or female?	
Experience and training	5	What experience or training did the researcher have?	
Relationship with participants			
Relationship established	6	Was a relationship established prior to study commencement?	
Participant knowledge of the interviewer	7	What did the participants know about the researcher? e.g. personal goals, reasons for doing the research	
Interviewer characteristics	8	What characteristics were reported about the inter viewer/facilitator? e.g. Bias, assumptions, reasons and interests in the research topic	
**Domain 2: Study design**			
Theoretical framework			
Methodological orientation and Theory	9	What methodological orientation was stated to underpin the study? e.g. grounded theory, discourse analysis, ethnography, phenomenology, content analysis	
Participant selection			
Sampling	10	How were participants selected? e.g. purposive, convenience, consecutive, snowball	
Method of approach	11	How were participants approached? e.g. face-to-face, telephone, mail, email	
Sample size	12	How many participants were in the study?	
Non-participation	13	How many people refused to participate or dropped out? Reasons?	
Setting			
Setting of data collection	14	Where was the data collected? e.g. home, clinic, workplace	
Presence of non-participants	15	Was anyone else present besides the participants and researchers?	
Description of sample	16	What are the important characteristics of the sample? e.g. demographic data, date	
Data collection			
Interview guide	17	Were questions, prompts, guides provided by the authors? Was it pilot tested?	
Repeat interviews	18	Were repeat inter views carried out? If yes, how many?	
Audio/visual recording	19	Did the research use audio or visual recording to collect the data?	
Field notes	20	Were field notes made during and/or after the inter view or focus group?	
Duration	21	What was the duration of the inter views or focus group?	
Data saturation	22	Was data saturation discussed?	
Transcripts returned	23	Were transcripts returned to participants for comment and/or	

For peer review only

Topic	Item No.	Guide Questions/Description	Reported on Page No.
		correction?	
**Domain 3: analysis and findings**			
Data analysis			
Number of data coders	24	How many data coders coded the data?	
Description of the coding tree	25	Did authors provide a description of the coding tree?	
Derivation of themes	26	Were themes identified in advance or derived from the data?	
Software	27	What software, if applicable, was used to manage the data?	
Participant checking	28	Did participants provide feedback on the findings?	
Reporting			
Quotations presented	29	Were participant quotations presented to illustrate the themes/findings? Was each quotation identified? e.g. participant number	
Data and findings consistent	30	Was there consistency between the data presented and the findings?	
Clarity of major themes	31	Were major themes clearly presented in the findings?	
Clarity of minor themes	32	Is there a description of diverse cases or discussion of minor themes?	

Developed from: Tong A, Sainsbury P, Craig J. Consolidated criteria for reporting qualitative research (COREQ): a 32-item checklist for interviews and focus groups. *International Journal for Quality in Health Care*. 2007. Volume 19, Number 6: pp. 349 – 357

Once you have completed this checklist, please save a copy and upload it as part of your submission. DO NOT include this checklist as part of the main manuscript document. It must be uploaded as a separate file.

For peer review only

BMJ Open

What is Multidisciplinary Cancer Care Like in Practice? A protocol for a mixed-method study to characterise ambulatory oncology services in the Australian public sector

Journal:	*BMJ Open*
Manuscript ID	bmjopen-2019-031179.R2
Article Type:	Protocol
Date Submitted by the Author:	06-Sep-2019

Complete List of Authors:	Nic Giolla Easpaig, Bróna; Macquarie University, Australian Institute of Health Innovation<br>Arnolda, Gaston; Macquarie University, Australian Institute of Health Innovation<br>Tran, Yvonne ; Macquarie University, Australian Institute of Health Innovation<br>Bierbaum, Mia; Macquarie University, Australian Institute of Health Innovation<br>Lamprell, Klay; Macquarie University, Australian Institute of Health Innovation<br>Delaney, Geoffrey; Liverpool Cancer Therapy Centre; University of New South Wales South Western Sydney Clinical School<br>Liauw, Winston; Saint George Hospital Saint George Cancer Care Centre; University of New South Wales, St George Hospital Clinical School<br>Chittajallu, Renuka; Macquarie University, Australian Institute of Health Innovation<br>Winata, Teresa; Macquarie University, Australian Institute of Health Innovation; Griffith University,<br>Ward, Robyn; University of Sydney, Faculty of Medicine and Health; University of New South Wales, Prince of Wales Clinical School<br>Currow, David ; Flinders University, College of Medicine and Public Health; University of Technology Sydney, Faculty of Health<br>Olver, Ian; University of South Australia, Division of Health Sciences<br>Karnon, Jonathan; Flinders University, College of Medicine and Public Health<br>Westbrook, Johanna; Macquarie University, Australian Institute of Health Innovation<br>Braithwaite, Jeffrey; Macquarie University, Australian Institute of Health Innovation

**Primary Subject Heading**:	Health services research
Secondary Subject Heading:	Oncology, Health services research, Patient-centred medicine, Health economics, Evidence based practice
Keywords:	cancer, QUALITATIVE RESEARCH, multi-site, ONCOLOGY, outpatient clinic

SCHOLARONE™
Manuscripts

Title: What is Multidisciplinary Cancer Care Like in Practice? A protocol for a mixed-method study to characterise ambulatory oncology services in the Australian public sector

Authors:

1. Nic Giolla Easpaig, Bróna (Corresponding author)

   Postal Address: Australian Institute of Health Innovation, Macquarie University Level 6, 75 Talavera Road, Sydney, NSW 2109, Australia

   Email: brona.nicgiollaeaspaig@mq.edu.au

   Phone number: +61 2 9850 2400

2. Arnolda, Gaston

   Australian Institute of Health Innovation, Macquarie University, Sydney, NSW, Australia

3. Tran, Yvonne

   Australian Institute of Health Innovation, Macquarie University, Sydney, NSW, Australia

4. Bierbaum, Mia

   Australian Institute of Health Innovation, Macquarie University, Sydney, NSW, Australia

5. Lamprell, Klay,

   Australian Institute of Health Innovation, Macquarie University, Sydney, NSW, Australia

6. Delaney, Geoffrey, P.

   i. Liverpool Cancer Therapy Centre, Liverpool, NSW, Australia

For peer review only - http://bmjopen.bmj.com/site/about/guidelines.xhtml

For peer review only

ii. South Western Sydney Clinical School, University of New South Wales, Liverpool, NSW, Australia

7. Liauw, Winston,

   i. St. George Cancer Care Centre, St. George Hospital, Kogarah, NSW, Australia

   ii. St. George Hospital Clinical School, University of New South Wales, Sydney, NSW, Australia

8. Chittajallu, Renuka

   Australian Institute of Health Innovation, Macquarie University, Sydney, NSW, Australia

9. Winata, Teresa

   Australian Institute of Health Innovation, Macquarie University, Sydney, NSW, Australia

10. Ward, Robyn, L.

   i. Faculty of Medicine and Health, The University of Sydney, Sydney, NSW, Australia

   ii. Prince of Wales Clinical School, University of New South Wales, Sydney, NSW, Australia

11. Currow, David

   i. College of Medicine and Public Health, Flinders University, Adelaide, SA, Australia

   ii. Faculty of Health, University of Technology Sydney, Sydney, NSW, Australia

12. Olver, Ian,

   Division of Health Sciences, University of South Australia, Adelaide, SA, Australia

13. Karnon, Jon,

   College of Medicine and Public Health, Flinders University, Adelaide, SA, Australia

For peer review only - http://bmjopen.bmj.com/site/about/guidelines.xhtml

14. Westbrook, Johanna

Australian Institute of Health Innovation, Macquarie University, Sydney, NSW, Australia

15. Braithwaite, Jeffrey

Australian Institute of Health Innovation, Macquarie University, Sydney, NSW, Australia

Word count: *4,323* (excluding title page, abstract, references, figures and tables)

Keywords: qualitative research; multi-site; oncology; cancer; outpatient clinic

For peer review only

For peer review only - http://bmjopen.bmj.com/site/about/guidelines.xhtml

ABSTRACT

Introduction

An understanding of the real-world provision of oncology outpatient services can help maintain service quality in the face of escalating demand and tight budgets, by informing the design of interventions that improve the effectiveness or efficiency of provision. The aims of this study are threefold. First, to develop an understanding of cancer services in outpatient clinics by characterising the organisation and practice of multidisciplinary care (MDC). Second, to explore the key areas of: a) clinical decision-making; and b) engagement with patients' supportive needs. Third, to identify barriers to, and facilitators of, the delivery of quality care in these settings.

Methods and analysis

A suite of mixed-methods studies will be implemented at six hospitals providing cancer outpatient clinics (OPCs), with a staged roll-out. In Stage One, we will examine policies, use unstructured observations and undertake interviews with key health professionals to characterise the organisation and delivery of MDC. In Stage Two, observations of practice will continue, to deepen our understanding, and to inform two focused studies. The first will explore decision-making practices and the second will examine how staff engage with patients' needs; both studies involve interviews, to complement observation. As part of the study of supportive care, we will examine the implications of an introduction of patient-reported measures (PRMs) into care, adding surveys to interviews before and after PRMs roll-out. Data analysis will account for site-specific and cross-site issues using an adapted Qualitative Rapid Appraisal, Rigorous Analysis approach. Quantitative data from clinician surveys will be statistically analysed and triangulated with the related qualitative study findings.

Ethics and dissemination

For peer review only - http://bmjopen.bmj.com/site/about/guidelines.xhtml

Ethical approval was granted by South Eastern Sydney Local Health District Human Research Ethics Committee (no. 18/207). Findings will be shared with participating hospitals and widely disseminated through publications and presentations.

For peer review only

For peer review only - http://bmjopen.bmj.com/site/about/guidelines.xhtml

STRENGTHS AND LIMITATIONS OF THIS STUDY

- Multiple data types and sources are integrated to characterise MDC in different settings.
- A two-stage research design facilitates better targeting of observations and interviews in the second stage.
- A strategy of intensive data collection using multiple methods is used to ensure that site-specific data is interpreted within its local context.
- Findings will be based on a rigorous and extensive observational characterisation of Australian oncology outpatient services.

For peer review only

For peer review only - http://bmjopen.bmj.com/site/about/guidelines.xhtml

INTRODUCTION

In the developed world, invasive cancer is the leading cause of death.[1] Globally, the incidence of cancer is increasing;[2] in Australia, cancer incidence is projected to reach 150,000 new cases per year by 2020.[3] Survival rates are improving, with over 400,000 Australians living 5 years post cancer diagnosis.[3] Cancer is understood as a condition requiring medical and supportive care services from diagnosis through long-term survivorship.[4,5] Multidisciplinary care (MDC) is accepted as a best practice model of care provision for oncology services in Australia, and in advanced health systems globally.[6-9] MDC seeks to promote equitable, evidence-based care by a team that combines relevant expertise and enables patients to be involved in decision-making concerning their care.[5,10]

Work has been undertaken to translate MDC principles into practice; but significant challenges remain.[10-12] In this regard, substantial evidence points to gaps between optimal evidence-based care and current care.[13-17] A review of research literature, national health sector reports and local cancer plans identified three key interconnected elements of MDC: diverse professional expertise; clinical decision-making; and, addressing patients' supportive care needs. In Figure 1 the coordination of care is positioned to reflect the way in which it binds and encompasses these elements.

<INSERT FIGURE 1. HERE>

Source: Authors' conceptualisation of MDC

Patient needs

In addition to medical anti-cancer treatment with curative intent, people with cancer also need medical (e.g., pain relief) and non-medical supportive care (e.g., information needs). The challenges of addressing supportive needs are an ongoing concern in oncology.[18-21]

For peer review only - http://bmjopen.bmj.com/site/about/guidelines.xhtml

Meeting these needs requires identification, prioritisation, timely access to relevant professional expertise and treatment planning (e.g. in consultation with a psychologist).[18-21] In general, identification of support needs in cancer care has not been systematic. Rather, routine care has relied on the clinical acumen of individual clinicians who identify needs during consultations and make appropriate referrals. Language spoken and level of health literacy also remain challenges in order to "pitch" information at the level most suitable for each patient. Routinely collecting information from patients, through Patient Reported Measures (PRMs), has been promoted as a mechanism to identify unmet supportive needs.[22-24] The Edmonton Symptom Assessment Scale[25], Distress Thermometer[26] are PRMs which some of the outpatient clinics (OPCs) will be introducing into routine cancer care during the period of study, and are expected to change the management of patients' supportive needs.[27-29] This study focuses on the provider perspective; the patient's support networks are also important for addressing supportive needs, but these are beyond the study's scope.

Diverse professional expertise

MDC is overseen by a core team which, while varying by cancer type, usually consists of pathologists, radiologists, medical oncologists, radiation oncologists, surgeons, nurses and allied health professionals.[5,10,30] Other specialist expertise can also be part of the core team, or can be added as required. This may include expertise such as in palliative care or pre- and rehabilitation over the course of the patient's journey.[5] This approach is intended to utilise cross-disciplinary expertise to the fullest benefit of the patient and offer shared decision-making mechanisms for clinicians.[10,31] To develop an understanding of MDC we therefore need to harness the views of a diverse range of professionals and observe interprofessional collaboration in practice.

For peer review only - http://bmjopen.bmj.com/site/about/guidelines.xhtml

Decision-making

Multidisciplinary team meetings (MDTMs) are a fulcrum for the clinical coordination of MDC.[11,12] Research suggests that MDTM discussions influence diagnosis, staging and treatment planning.[32-34] It is important to understand the organisation of MDTMs and the resources required to support them. Studies reveal variability in the organisation of MDTMs, who is discussed and when, and the ways that clinicians engage to support clinical decision-making.[9] While MDTMs play a central role, clinicians make decisions outside MDTMs, without any consultation during routine care provision, or in consultation with other clinicians who may or may not be part of the team. In addition, decisions can be made at the meetings but evidence suggests that the recommendations are not always followed and in many meetings there can be disagreements in opinion.[35] How these issues are managed with the patient are likely to be highly individualised. We also seek to understand this behaviour.

Coordinated care

Among the top priorities for coordination are: 1) ongoing screening of patients' supportive needs and appropriate care in response and 2) the timely and appropriate delivery of care across the patient's journey.[36] Public hospital OPCs are tasked with providing care for diverse types of cancer across the patient journey, from diagnosis, through treatment and into survivorship. The models of coordination employed may be non-uniform; hybrid approaches may be used within a setting (e.g., simultaneously offering tumour stream-specialised care coordination, with generalised care coordinators covering all others).[37,38] Sub-optimal coordination can lead to fragmented care, conflicting information provided to patients, impaired access to appropriate services and inefficient use of the time and energy of health professionals and patients.[21,39,40]

For peer review only - http://bmjopen.bmj.com/site/about/guidelines.xhtml

Research framework

The investigation described in this paper is the first major phase of field research undertaken by the newly established National Health and Medical Research Council (NHMRC) Centre for Research Excellence in Implementation Science in Oncology (CRE-ISO), administered by the Australian Institute for Health Innovation at Macquarie University. In healthcare, implementation science focuses on understanding processes and methods for supporting the uptake and integration of effective interventions to improve healthcare practices and outcomes.[41] The research described here will characterise MDC provision in two Sydney metropolitan Local Health Districts (LHDs), government agencies responsible for managing and providing public health services within a specified geography, usually of approximately one million residents in order to provide a foundational understanding of the realities of oncology service provision. This understanding will inform subsequent phases of research undertaken by the Centre and will guide the selection of implementation approaches, some of which will focus on developing and testing tailored interventions to address identified evidence-practice gaps.

Critical to this study is engagement with the context of service provision and its complexity; of the "interacting and interdependent" components of a health service that comprise MDC provision in hospitals.[42] Multiple sources of data will be harvested and integrated, exploring the formalised processes and local practices undertaken within the interconnected networks of semi-autonomous professionals that together shape care provision.[43] Systems of care provision in these settings are rarely static, and opportunities will be taken to examine the uptake of new practices, such as the introduction of PRMs, to learn about the processes that inhibit or promote effective adoption. This research will also seek to identify generic barriers and facilitators that impede or promote best practice, as background information to assist with the design of future interventions.

For peer review only - http://bmjopen.bmj.com/site/about/guidelines.xhtml

Research Aims (RA)

The study aims to:

RA1. Develop understanding of cancer services in OPCs by characterising the organisation and practice of MDC;

RA2. Explore, in-depth, selected key elements of MDC:

i. Clinical decision-making in MDC and how doctors engage with decision-making support mechanisms in practice; and

ii. How health professionals engage with patients' supportive needs before and immediately after the introduction of PRMs.

RA3. Identify general barriers to, and facilitators of, the provision of MDC.

For peer review only

METHODS & ANALYSIS

A mixed-methods research design will be adopted: largely comprised of a variety of qualitative methods, complimented by a single quantitative survey. Methods adapted from ethnography will be harnessed, to facilitate intensive and efficient data collection; rapid ethnography will be used to reduce the burden of research on organisations and participants while maintaining rigour and richness.[44,45] Careful thought has been given to the demands of multi-site research and we adopt recommended strategies used to enhance validity and maintain capacity to adequately engage with the complexities of the settings.[46]

The methodological principles embedded in the design are: exploring everyday practices and the organisational relations that shape them, by observing participants at work in OPC settings; harnessing a plurality of participant perspectives, by interviewing professionals about their work; and generating rich descriptive accounts of MDC contexts by using analytical and interpretative approaches formulated for multi-site data.[47] A critical realist philosophical position is taken, which is comprised of a realist ontological assumption and a critical epistemology.[48,49] From this position it is assumed that a given account may not perfectly reflect reality; rather, accounts may be contextually and structurally mediated, offering partial insight into complex phenomena.[50,51]

Design

A staged design will be used. Prior to study commencement, profiles and structural descriptions of each hospital setting were developed, using publicly-available information. In *Stage One,* an overview of the organisation of practice and models of care will be derived through interviews with “Navigators” (e.g., OPC staff with an overview of care pathways such as Nurse Unit Managers or care coordinators), review of unpublished policies and procedures and unstructured observations of care provision. In *Stage Two*, we enrich the accounts of these

For peer review only - http://bmjopen.bmj.com/site/about/guidelines.xhtml

settings through additional observation in OPCs. Together, these methods address the first research aim (RA1) and provide a foundation for the second (RA2) and third (RA3).

Stage Two also includes focused studies, undertaken in parallel. In Study 2.1, interviews with doctors and observations of cancer MDTMs provide insight into clinical decision-making practices in MDC and how doctors engage with decision-making support mechanisms (RA2.i). Engagement with patients' supportive needs will be explored in Study 2.2 (RA2.ii), using a two part, mixed-method design consisting of interviews with health professionals, observation of cancer MDTMs and a "clinician readiness" survey; all research elements will be performed before introduction of new PRMs, and repeated after they have been introduced.

Barriers and facilitators to the provision of MDC (RA3) will be elicited as part of the interviews in each study and enhanced by our observational studies in *Stages One* and *Two*. The study design is informed by the Consolidated Criteria for Reporting Qualitative Research.[52] The study will run from the 28th of February, 2019 up to the 31st of December 2020.

Selection of sites and participants

The investigation will be undertaken within two metropolitan LHDs, each containing three participating public hospitals with OPCs. Collectively, the OPCs offer an extensive range of consultation, coordination, wellness and outpatient treatment services. Each LHD contains two large hospitals which provide an extensive range of services and a smaller hospital offering a more limited range of services, closely linked with larger centres (e.g., including the provision of teleconferencing for meetings).

Sites and meetings: In *Stage One* all sites will be studied, to understand each hospital setting. In *Stage Two*, maximum variation sampling[53] will be used to allocate the 'continuing observation' sessions to participating sites. As there are differences in the services between sites (e.g. some hospitals offer a smaller range), observation sessions will be allocated to best

For peer review only - http://bmjopen.bmj.com/site/about/guidelines.xhtml

capture the diverse range of practices and processes useful in the characterisation of MDC within and across sites. Purposive sampling will be used to select the MDTMs approached to participate. We will generate a sample comprising a range of tumour streams including common and rarer cancers. Study 2.1 will largely be conducted in one LHD, with study 2.2 in the other.

Participants: The participant inclusion criteria for interviews, surveys and health professional observations is generally broad: core medical staff (e.g., medical oncologists, surgeons, radiation oncologists); nursing staff (e.g., clinical nurse consultants, cancer care coordinators); and allied health staff (e.g., social workers, psychologists). However, participation in *Stage One* “Navigator interviews” will be restricted to staff with an overview of care pathways (e.g., cancer care coordinators, Nurse Unit Managers, managers of clinical streams). Similarly, in Study 2.2 the “clinician decision-making” interviews will be undertaken with specialist doctors (e.g. medical oncologists, surgeons, radiation oncologists) working in cancer services.

Recruitment: Information about the research including its aims, an introduction to the researchers undertaking data collection, contact details and participant information sheets will be disseminated through email, flyers and posters, and through information sessions for target professional groups at each site. Snowball sampling will also be used; participants will be encouraged to discuss the project with colleagues. Letters will be sent to the Chairpersons of cancer MDTMs and to the authorities responsible for the OPCs, to provide information about the research and request permission for the observations. These letters will include “Health Professional Information Sheets” which recipients will be encouraged to share with relevant colleagues as a component of pre-approval consultation.

Sample size ranges: The Study design features, presented in Table 1, summarise the stages of the research, the data collected in each stage and the target number of observations,

For peer review only - http://bmjopen.bmj.com/site/about/guidelines.xhtml

interviews and surveys. The sample sizes designated for the interview components are guided by principles identified in reported research literature, as well as Malterud, Siersma and Guassora's (2016) model for generating "information power" in qualitative studies. The model accounts for the following factors when estimating a sample range: the study aim; specificity of the sample; the envisioned quality of the interview interaction; analysis strategy; and established theory.[54] The number of proposed interviews in Study 2.2. is also guided by the sample size needed for the clinician readiness survey that is undertaken as part of the interview. The sample size allocated for the individual health professional observations reflects the narrow and specific focus of gaining a deep understanding of the roles these professionals play in MDC. The number of sessions proposed for the observations of the OPCs and MDTMs are comparable with similar qualitative studies[55] and are appropriate for multi-site ethnographic studies.[56]

Research Team: The investigation will be overseen by a team with extensive experience in health services and quality improvement research, including leaders in implementation science and oncology. Fieldwork will be guided by principal investigators with direct clinical and research experience in oncology service provision, in leadership roles in the LHDs. Data will be collected by GA (Senior Research Fellow, Ph.D., male), BNGE (Research Fellow, Ph.D., female) and TW (Research Associate, Masters, female) who are trained in qualitative, quantitative and mixed-methods investigation. Each of these researchers has fieldwork experience across a range of health services. Qualitative analysis will be conducted by BNGE and TW, while YT (Research Fellow, Ph.D., female) will analyse quantitative data. Appropriately qualified health professionals who join the research team for *Stage Two* will be trained and supervised.

For peer review only - http://bmjopen.bmj.com/site/about/guidelines.xhtml

Table 1. Study design features

Phase/Stage	Text	Interviews	Survey	MDTM Observations	Undirected Observation Sessions*
**Stage One**					
**Stage One Multi-site characterisation**	Review of policies and procedures relevant to interview discussion	• Navigator (n=8-16)<br>• Decision-making (n=3-8)<br>• Supportive needs (n=4-10)	n/a	Cancer MDTMs (n=3-8)	Initial:<br>• OPCs (n=15-25)<br>• Individual (n=3-6)
**Stage Two**					
**Study 2.1 Decision-making**	Review of policies and procedures relevant to interview discussion	Decision-making (n=15-30)	n/a	Cancer MDTMs (n=3-8)	Ongoing:<br>• OPCs (n=15)<br>• Individual (n=7)
**Study 2.2. Supportive needs**		• Baseline (n=15-30)<br>• Follow-up (n=15-30)	• Baseline (n=30-50)<br>• Follow-up (n=30-50)	Cancer MDTMs (n=3-8)	
**Estimated totals**	n/a	60- 124	30-50	9-24	• OPCs (n=30-40)<br>• Individual (n=10)

Table key

	Undertaken at all sites
(blue)	Predominantly undertaken in LHD 1
(green)	Predominantly undertaken in LHD 2
*	1 observation session= half day or 3-4hrs

Legend: MDTM=Multidisciplinary Team Meeting; OPC=cancer outpatient clinic.

* 1 observation session= half day or 3-4hrs

For peer review only

For peer review only - http://bmjopen.bmj.com/site/about/guidelines.xhtml

Data Collection

As part of protocol development, reviews of the literature were conducted to conceptualise and identify key issues in MDC provision and to scope the methodological scholarship relevant to the design. Publicly-available annual reports and cancer plans from each health district were analysed to generate a description of cancer services at each hospital. These profiles were used to map key areas for data collection. Implementation science approaches emphasise the importance of the local context of adoption and engagement with its stakeholders.[41-43] During this stage, input was sought from oncology management to ensure the local relevance of the research and the feasibility of the proposed methods.

Stage One. Multi-site characterisation study

Interviews: Semi-structured "Navigator" interviews will be undertaken with professionals who have overview roles (e.g., cancer care coordinator), oriented by care pathway maps, to establish the models of care and processes involved in the patient journey (n=8-16).[57,58] The care pathway maps were derived from the stages of MDC depicted by Fennell, Das, Clauser, Petrelli and Salner (2010),[59] focused on the diagnosis and OPC treatment stages (Figure 2). Through the interviews, maps will be reworked to reflect the Australian services context, and the differing trajectories followed by different: a) tumour streams (accounting for tumour-specific services); and b) risk categories and complexity. Where interviewees refer to specific policies and procedures, documentation will be formally requested to help situate the discussion within its policy context. Interviews are expected to take approximately 40 minutes and will be audio-recorded and transcribed.

Observations: Three types of observations will be made in the research:

- Unstructured observations of OPCs will provide an understanding of the real-world, routine organisation and actual delivery of MDC in these settings (e.g., we will observe *ad hoc* and

For peer review only - http://bmjopen.bmj.com/site/about/guidelines.xhtml

planned collaboration between professionals from differing disciplines during the care delivery process; n=15-25).

- Observations of individual health professionals will capture the key activities undertaken, to understand how their work contributes to the provision of MDC (n=3-6 individual health professionals observed for half or all of their day).

- Observations of MDTMs will be conducted using a semi-structured template (Figure 3) informed by previous MDTM observational research to guide what we observe and record, and systematise our description of meetings (n=3-8 meetings).[11,60] Fieldnotes will record observations of: professional groups in attendance; specific roles; support provided to administer the meeting (i.e. technology); organisation of cases; clinical decision-making; patient-centred care, and; organisation of follow-up.

All observations will be recorded in hand-written fieldnotes which will be used to generate rich accounts of care.

<INSERT FIGURE 2. HERE>

Source: Fennell et al. (2010)[59] (adapted from Zapka et al., 2003)[61]

Transition to Stage Two: During *Stage One*, we will pilot elements of the clinician interviews about decision-making (n=3-8) and patients' supportive needs (n=4-10) to be undertaken in *Stage Two,* and interview prompts will be refined. Where eligible health professionals indicate interest in joining the research team for *Stage Two* fieldwork, they will be briefed and provided with necessary training and support.

For peer review only - http://bmjopen.bmj.com/site/about/guidelines.xhtml

<INSERT FIGURE 3: HERE>

Source: Developed by authors based on Harris et al (2016)[60] and Rankin et al. (2018)[11]

Stage Two. MDC Studies

This stage will enrich accounts of MDC provision via "ongoing characterisation" (i.e. n=3-6 further observations of individual health and n=15 unstructured observations of OPCs), at the same time contextualising the focused studies detailed below.

Study 2.1 Clinical decision-making

Interviews: Doctors will participate in semi-structured interviews that explore their decision-making practices and their engagement with support mechanisms (n=15-30). Topics will include their perceptions of decision-making mechanisms, the decision-making support available to them, their own decision-making practices and their use of MDTMs at key points in the patient journey. A conceptual map of the patient journey (Figure 4) will be used to orient the interview, with questions tailored to each stage. Barriers to and drivers of efficient clinical decision-making will be explored. Interviews are expected to take approximately 40 minutes and will be audio-recorded and transcribed.

<INSERT FIGURE 4. HERE>

Source: Authors' adaption of Fennell et al. (2010)[59]

For peer review only - http://bmjopen.bmj.com/site/about/guidelines.xhtml

Observation of MDTMs: Meetings will be observed using a semi-structured template (Figure 3), focussing on the "clinical decision-making" component (n=3-8). Observational data will capture information about this decision-making mechanism and contextualise interviews.

Study 2.2 Patients' supportive needs

Interviews: Prior to introduction of PRMs, semi-structured interviews will be conducted with health professionals about how they engage with patients' supportive needs in their current practice (n=15-30). Topics will include: models of care; their individual role; identification and screening; management; and any barriers and facilitators experienced in identifying and responding to supportive needs. The interviews will also explore preparations for and expectations of PRMs.

Following the adoption of PRMs, health professionals will again participate in semi-structured interviews (n=15-30). These will focus on the adoption and implications of PRMs in relation to the topics addressed in pre-introduction interviews. Pre- and post-introduction interviews are expected to take approximately 40 minutes and will be audio-recorded and transcribed.

Surveys: An eight-item clinician readiness survey[62] will be completed by health professionals before and after the adoption of PRMs (n=30-50 in each phase). The survey will assess the current level of preparedness, and changes over time. This survey will take approximately five minutes to complete and will be performed as part of the interviews and will also be distributed more widely to achieve the desired sample size.

Observation of MDTMs: Meetings will be observed using a semi-structured template (Figure 3), focussing on the "patient-centred care & supportive care needs" component (n=3-8). Data will capture information about engagement with supportive needs in MDTMs and contextualise the interviews.

For peer review only - http://bmjopen.bmj.com/site/about/guidelines.xhtml

Data Analysis

Interview data: Basic descriptive data collected during interviews (e.g. listing of team members involved in different points of the patient journey) will be collated across interviews then categorised and organised within a matrix. The discursive data will be coded by two researchers and thematically analysed, oriented by an inductive approach.[63] Where process maps are used (e.g. navigator and decision-making interviews), these will frame the analysis. Constant comparative analysis will be applied to interpret data gathered before and after the introduction of PRMs. This strategy accounts for the cross-site, multiple-source nature of the data. Our approach for making sense of cross-site data is informed by the "Qualitative Rapid Appraisal, Rigorous Analysis" methodology developed by Phillips et al. (2014).[45] First, all "like" data (e.g., all interview data) is analysed at a site, followed by intra-site analysis of all data from one OPC. Inter-site analysis is then employed to consider the data across the sites.[45]

Observational data: Fieldnotes will be indexed to identify the specific setting/MDTM meeting, key processes and events and study focus. The notes will be integrated within hospital cancer service profiles to elaborate descriptions of OPC settings (i.e., the organisational features of care). Fieldnotes from MDTM and OPC observations will then be developed into accounts that contextualise data collected in the focused studies. Data collected from individual health professional observations will be developed into an account of the role.

Clinician readiness survey data: Analysis will include a repeated-measures ANOVA to test for differences in clinician attitudes before and after PRMS roll-out. If possible, subgroup analyses will test for differences in attitudes between different demographic and clinical subgroups. The quantitative data will be used to complement the interview data on this topic. Based on known effect sizes from results of the Willis et al. (2009)[62] paper using the Outcome Measurement Questionnaire to assess change in response to training, using a power of 0.8 and alpha of 0.05, the minimum sample size was estimated as n=32 participants (i.e., to

For peer review only - http://bmjopen.bmj.com/site/about/guidelines.xhtml

detect a change in mean scale score from 4.09 pre- to 4.49 post-introduction, with a standard deviation of 0.57).

Measurement of outcomes

The main outcomes of the investigation are aligned with the aims. Through this investigation we will generate a rich and comprehensive description of the organisation and practice of MDC. We will document practices of clinical decision-making including the role of the MDT in such practice. We will produce an account of how patient supportive needs are engaged with and the implications that the introduction of PRMs has for this engagement. The factors which impede or promote practice will be identified and used to inform subsequent CRE-ISO activity.

For the qualitatively-driven research, assessment of the outcomes involves judgement of the adequacy with which the research aims were addressed. This is largely reliant on the quality of the data collected and the rigour of its analysis. Sufficiently detailed data are needed from a range of sources to produce rich descriptions of MDC practice, including its diversity. Fieldwork and analytic strategies should allow findings to be situated within OPC contexts as well as revealing points of connection and contradiction between sites. Characterisation of MDC provision will be achieved through credible, adequately complex and well-grounded accounts. Multi-method data collection (including a quantitative component) allows for consideration of various sources of input, and triangulation; a team-based approach to analysis will facilitate ongoing reflection and monitoring.

For peer review only - http://bmjopen.bmj.com/site/about/guidelines.xhtml

ETHICS & DISSEMINATION

Ethical approval was granted by South Eastern Sydney LHD Human Research Ethics Committee (#18-207). Informed written consent will be obtained from interviewees and from health professionals who are individually observed. Interview and observation arrangements will be made with health professionals at least one week in advance to ensure they have time to prepare. Participants may withdraw, without justification, up to one-week post interview/observation or whenever the data is de-identified.

Posters will be displayed, and information sheets will be available during observational sessions so that those present can raise concerns or decline participation. This can be done at any time up until the observational data is collected. The research team will not collect any information about patients and, guided by health professionals, will minimise exposure to direct patient interactions. Researchers will answer questions posed about their presence and provide information sheets that contain ethics committee details. In the event of participants experiencing discomfort or distress arising from the research, researchers will facilitate access to appropriate support services.

Data storage and protection: Data will be stored on a password-protected computer and on a secure University server, accessible only to the research team. Data will be de-identified and separated from participant identifiers. Research records will be retained for at least five years post-study completion or last publication, in accordance with Section 2.1.1 of the Australian Code for the Conduct of Responsible Research (2007)[64] before secure destruction.

Dissemination: Findings will be disseminated to academics, professionals and the public through publications and presentations. Tailored reports will be provided to participating hospitals and presentations given to participating professional groups.

For peer review only - http://bmjopen.bmj.com/site/about/guidelines.xhtml

Patient and Public Involvement Statement

The coordinating research institution routinely consults with patients, their representatives and the general public, to ensure that adequate input is secured for research programs; a key partner is the Consumers Health Forum of Australia. This specific project was developed without detailed patient involvement; patients were not invited to comment on the study design or to the writing or editing of this protocol. This project will help us to describe service structure and organisation, and to learn from the perspectives of MDC providers. The experiences of patients are vital to understanding these services, including their views on the barriers and facilitators to high-quality care. Patient experiences will be engaged with in dedicated, patient-centred studies within the CRE-ISO program of research. For example, in preparation, we have already looked at data from UK Patient Experience Surveys[65], and are in the process of accessing Australian data from a similar source.

For peer review only

For peer review only - http://bmjopen.bmj.com/site/about/guidelines.xhtml

IMPACT & SIGNIFICANCE

The research seeks to capture a diverse range of practices and processes useful in the characterisation of MDC, with attention to site-specific nuances that may be relevant to future improvement efforts. The accumulative characterisation will be combined with focused, in-depth studies of clinical decision-making and processes for meeting patients' supportive care needs. The research involves rigorous and extensive observations of Australian public oncology OPCs and MDTMs; it offers a substantial opportunity for the generation of new and critical insights. The findings will serve as a foundation for the design of studies to address identified evidence-practice gaps in cancer care.

For peer review only

References

1. Jemal A, Bray F, Center MM, et al. Global cancer statistics. *CA Cancer J Clin* 2011;61:69-90. doi:10.3322/caac.20107

2. International Agency for Research on Cancer. GLOBOCAN 2018 Global Cancer Observatory, 2018. https://gco.iarc.fr/ (accessed 4 July 2019).

3. Australian Institute of Health and Welfare. Cancer incidence projections, Australia 2011 to 2020. Canberra, Australia: AIHW, 2012.

4. Australian Institute of Health and Welfare. Cancer in Australia. Canberra, Australia: AIHW, 2017.

5. Cancer Australia. All about multidisciplinary care. https://canceraustralia.gov.au/clinical-best-practice/multidisciplinary-care/all-about-multidisciplinary-care (accessed 20 July 2018).

6. American College of Surgeons: Commission on Cancer. Cancer program standards: ensuring patient-centered care. Chicago, IL: American College of Surgeons, 2016.

7. Jiang H. Patients should surely be included in multidisciplinary team meetings in China healthcare. *BMJ* [eLetter] 14 October 2015. https://www.bmj.com/content/351/bmj.h5295/rr-1

8. Pan C-C, Kung P-T, Wang Y-H, et al. Effects of multidisciplinary team care on the survival of patients with different stages of non-small cell lung cancer: a national cohort study. *PloS One* 2015;10:e0126547-e47. doi:10.1371/journal.pone.0126547

9. Saini KS, Taylor C, Ramirez AJ, et al. Role of the multidisciplinary team in breast cancer management: results from a large international survey involving 39 countries. *Ann Oncol* 2012;23:853-59. doi:10.1093/annonc/mdr352

For peer review only - http://bmjopen.bmj.com/site/about/guidelines.xhtml

10. Zorbas H, Barraclough B, Rainbird K, et al. Multidisciplinary care for women with early breast cancer in the Australian context: what does it mean? *Med J Aust* 2003;179:528-31.

11. Rankin NM, Lai M, Miller D, et al. Cancer multidisciplinary team meetings in practice: results from a multi-institutional quantitative survey and implications for policy change. *Asia Pac J Clin Oncol* 2018;14:74-83. doi:10.1111/ajco.12765

12. Wilcoxon H, Luxford K, Saunders C, et al. Multidisciplinary cancer care in Australia: a national audit highlights gaps in care and medico-legal risk for clinicians. *Asia Pac J Clin Oncol* 2011;7:34-40. doi:10.1111/j.1743-7563.2010.01369.x

13. Barton MB, Jacob S, Shafiq J, et al. Estimating the demand for radiotherapy from the evidence: a review of changes from 2003 to 2012. *Radiol Oncol* 2014;112:140-44. doi:10.1016/j.radonc.2014.03.024

14. Batumalai V, Shafiq J, Gabriel G, et al. Impact of radiotherapy underutilisation measured by survival shortfall, years of potential life lost and disability-adjusted life years lost in New South Wales, Australia. *Radiol Oncol* 2018;129:191-95. doi:10.1016/j.radonc.2018.06.026

15. Delaney GP, Barton MB. Evidence-based estimates of the demand for radiotherapy. *Clin Oncol* 2015;27:70-76. doi:10.1016/j.clon.2014.10.005

16. Hanna TP, Shafiq J, Delaney GP, et al. The population benefit of evidence-based radiotherapy: 5-Year local control and overall survival benefits. *Radiol Oncol* 2018;126:191-97. doi:10.1016/j.radonc.2017.11.004

17. Telli ML, Jensen KC, Vinayak S, et al. Phase II study of gemcitabine, carboplatin, and iniparib as neoadjuvant therapy for triple-negative and BRCA1/2 mutation–associated breast cancer with assessment of a tumor-based measure of genomic instability: PrECOG 0105. *J Clin Oncol* 2015;33:1895-901. doi:10.1200/jco.2014.57.0085

For peer review only - http://bmjopen.bmj.com/site/about/guidelines.xhtml

18. Dilworth S, Higgins I, Parker V, et al. Patient and health professional's perceived barriers to the delivery of psychosocial care to adults with cancer: a systematic review. *Psychooncology* 2014;23:601-12. doi:10.1002/pon.3474

19. Rankin NM, Barron JA, Lane LG, et al. Psychosocial oncology services in New South Wales. *Aust Health Rev* 2011;35:156-63. doi:10.1071/AH08730

20. Walsh J, Young JM, Harrison JD, et al. What is important in cancer care coordination? a qualitative investigation. *Eur J Cancer Care* 2011;20:220-27. doi:10.1111/j.1365-2354.2010.01187.x

21. Walsh J, Harrison JD, Young JM, et al. What are the current barriers to effective cancer care coordination? a qualitative study. *BMC Health Serv Res* 2010;10:132. doi:10.1186/1472-6963-10-132

22. Basch E, Deal AM, Kris MG, et al. Symptom monitoring with patient-reported outcomes during routine cancer treatment: a randomized controlled trial. *J Clin Oncol* 2016;34:557-65. doi:10.1200/jco.2015.63.0830

23. Luckett T, Butow PN, King MT. Improving patient outcomes through the routine use of patient-reported data in cancer clinics: future directions. *Psychooncology* 2009;18:1129-38.doi:10.1002/pon.1545

24. Williams K, Sansoni J, Morris D, et al. Patient-reported outcome measures: literature review. Wollongong, Australia, Australian Health Services Research Institute, University of Wollongong, 2016.

25. Watanabe SM, Nekolaichuk CL, Beaumont C. The Edmonton Symptom Assessment System, a proposed tool for distress screening in cancer patients: development and refinement. *Psychooncology* 2012;21:977-85. doi:10.1002/pon.1996

For peer review only

For peer review only - http://bmjopen.bmj.com/site/about/guidelines.xhtml

26. National Comprehensive Cancer Network (NCCN). NCCN Clinical Practice Guidelines in Oncology (NCCN Guidelines®) for distress management 2016.

27. Cancer Institute NSW. Patient-reported measures (PRMs) 2018. https://www.cancer.nsw.gov.au/how-we-help/quality-improvement/patient-reported-measures (accessed 30 August 2018).

28. Girgis A, Delaney GP, Miller AA. Utilising ehealth to support survivorship care. *Cancer Forum* 2015; 39:86-89.

29. Girgis A, Durcinoska I, Levesque JV, et al. eHealth system for collecting and utilizing patient reported outcome measures for personalized treatment and care (PROMPT-Care) among cancer patients: mixed methods approach to evaluate feasibility and acceptability. *J Med Internet Res* 2017;19:e330. doi:10.2196/jmir.8360

30. Lamprell K, Arnolda G, Delaney GP, et al. The challenge of putting principles into practice: resource tensions and real-world constraints in multidisciplinary oncology team meetings. *Asia Pac J Clin Oncol* Published Online First: 21 May 2019. doi:10.1111/ajco.13166

31. National Breast Cancer Centre. Multidisciplinary care (MDC) in Australia: a national demonstration project in breast cancer. Camperdown, NSW, National Breast Cancer Centre, 2003.

32. Brunner M, Gore SM, Read RL, et al. Head and neck multidisciplinary team meetings: effect on patient management. *Head Neck* 2015;37:1046-50. doi:10.1002/hed.23709

33. Davies AR, Deans DAC, Penman I, et al. The multidisciplinary team meeting improves staging accuracy and treatment selection for gastro-esophageal cancer. *Dis Esophagus* 2006;19:496-503. doi:10.1111/j.1442-2050.2006.00629.x

For peer review only - http://bmjopen.bmj.com/site/about/guidelines.xhtml

34. Friedland PL, Bozic B, Dewar J, et al. Impact of multidisciplinary team management in head and neck cancer patients. *Br J Cancer* 2011;104:1246–1248. doi:10.1038/bjc.2011.92

35. Hamilton DW, Heaven B, Thomson RG, et al. Multidisciplinary team decision-making in cancer and the absent patient: a qualitative study. *BMJ Open* 2016;6:e012559. doi:10.1136/bmjopen-2016-01255934.

36. Shaw T, York S, White K, et al. Defining success factors to describe coordinated care in cancer. *Transl Behav Med* 2018;8:357-65. doi:10.1093/tbm/iby022

37. Eicher MRE, Marquard S, Aebi S. A nurse is a nurse? a systematic review of the effectiveness of specialised nursing in breast cancer. *Eur J Cancer* 2006;42:3117-26. doi:10.1016/j.ejca.2006.07.007

38. Porter-Steele J, Tjondronegoro D, Seib C, et al. 'Not one size fits all': a brief review of models of care for women with breast cancer in Australia. *Cancer Forum* 2017;41:13-19.

39. Kripalani S, Jackson AT, Schnipper JL, et al. Transitions of care at hospital discharge. *J Hosp Med* 2007;5:314-23. doi:10.1002/jhm.228

40. Weaver SJ, Jacobsen PB. Cancer care coordination: opportunities for healthcare delivery research. *Transl Behav Med* 2018;8:503-08. doi:10.1093/tbm/ibx07939.

41. Rapport F, Clay-Williams R, Churruca K, et al. The struggle of translating science into action: foundational concepts of implementation science. *J Eval Clin Pract* 2018;24:117-26. doi:10.1111/jep.12741

42. Braithwaite J, Marks D, Taylor N. Harnessing implementation science to improve care quality and patient safety: a systematic review of targeted literature. *Int J Qual Health Care* 2014;26:321-29. doi:10.1093/intqhc/mzu047

For peer review only - http://bmjopen.bmj.com/site/about/guidelines.xhtml

43. Braithwaite J, Churruca K, Long JC, et al. When complexity science meets implementation science: a theoretical and empirical analysis of systems change. *BMC Med* 2018;16:63. doi:10.1186/s12916-018-1057-z

44. Handwerker W. Quick Ethnography. Walnut Creek, CA: Altamira Press 2001.

45. Phillips CB, Dwan K, Hepworth J, et al. Using qualitative mixed methods to study small health care organizations while maximising trustworthiness and authenticity. *BMC Health Serv Res* 2014;14:559. doi:10.1186/s12913-014-0559-4

46. Millen D. Rapid ethnography: time deepening strategies for HCI field research. Proceedings of the 3rd Conference on Designing Interactive Systems: Processes, Practices, Methods, and Techniques; 2001 August 17-19; New York, USA: ACM, 2000. 45.

47. Charlesworth S, Baines D. Understanding the negotiation of paid and unpaid care work in community services in cross-national perspective: the contribution of a rapid ethnographic approach. *J Fam Stud* 2015;21:7-21. doi:10.1080/13229400.2015.1010263

48. Harper D. Choosing a qualitative research method. In: Harper D, Thompson AR, eds. Qualitative Research Methods in Mental Health and Psychotherapy: A Guide for Students and Practitioners. Hoboken, NJ: Wiley-Blackwell 2011: 83–98.

49. Bentall DPR. The medicalisation of misery: a critical realist analysis of the concept of depression. *J Ment Health* 1999;8:261-74. doi:10.1080/09638239917427

50. Harper D, Thompson AR. Qualitative Research Methods in Mental Health and Psychotherapy: A Guide for Students and Practitioners. Hoboken, NJ: Wiley-Blackwell 2011.

51. Willig C. Introducing Qualitative Research in Psychology. Maidenhead, UK: McGraw-Hill Education 2013.

For peer review only - http://bmjopen.bmj.com/site/about/guidelines.xhtml

52. Tong A, Sainsbury P, Craig J. Consolidated criteria for reporting qualitative research (COREQ): a 32-item checklist for interviews and focus groups. *Int J Qual Health Care* 2007;19:349-57. doi:10.1093/intqhc/mzm042

53. Palinkas LA, Horwitz SM, Green CA, et al. Purposeful sampling for qualitative data collection and analysis in mixed method implementation research. *Adm Policy Ment Health* 2015;42:533-44. doi:10.1007/s10488-013-0528-y

54. Malterud K, Siersma VD, Guassora AD. Sample size in qualitative interview studies: guided by information power. *Qual Health Res* 2015;26:1753-60. doi:10.1177/1049732315617444

55. Bridges J, Hughes J, Farrington N, et al. Cancer treatment decision-making processes for older patients with complex needs: a qualitative study. *BMJ Open* 2015;5:e009674. doi:10.1136/bmjopen-2015-009674

56. Renedo A, Marston C. Developing patient-centred care: an ethnographic study of patient perceptions and influence on quality improvement. *BMC Health Serv Res* 2015;15:122. doi:10.1186/s12913-015-0770-y55.

57. Lichtner V, Dowding D, Allcock N, et al. The assessment and management of pain in patients with dementia in hospital settings: a multi-case exploratory study from a decision making perspective. *BMC Health Serv Res* 2016;16:427. doi:10.1186/s12913-016-1690-1

58. Trebble TM, Hansi N, Hydes T, et al. Process mapping the patient journey: an introduction. *BMJ* 2010;341 doi:10.1136/bmj.c4078

59. Fennell ML, Prabhu Das I, Clauser S, et al. The organization of multidisciplinary care teams: modeling internal and external influences on cancer care quality. JNCI Monographs 2010;40:72-80. doi:10.1093/jncimonographs/lgq010

For peer review only - http://bmjopen.bmj.com/site/about/guidelines.xhtml

60. Harris J, Taylor C, Sevdalis N, et al. Development and testing of the cancer multidisciplinary team meeting observational tool (MDT-MOT). *Int J Qual Health Care* 2016;28:332-38. doi:10.1093/intqhc/mzw030

61. Zapka JG, Taplin SH, Solberg LI, et al. A framework for improving the quality of cancer care: the case of breast and cervical cancer screening. *Cancer Epidemiol Biomark Prev* 2003;12:4-13. 60.

62. Willis A, Deane FP, Coombs T. Improving clinicians' attitudes toward providing feedback on routine outcome assessments. *Int J Ment Health Nurs* 2009;18:211-15. doi:10.1111/j.1447-0349.2009.00596.x

63. Joffe H. Thematic analysis. In: Harper D, Thompson AR, eds. Qualitative Research Methods in Mental Health and Psychotherapy: A Guide for Students and Practitioners. Hoboken, NJ: Wiley-Blackwell 2011:209-223.

64. National Health Medical Research Council. Australian Code for the Responsible Conduct of Research: National Health and Medical Research Council, the Australian Research Council and Universities, Australia, 2007.

65. Tran Y, Lamprell K, Nic Giolla Easpaig B, et al. What information do patients want across their cancer journeys? a network analysis of cancer patients' information needs. *Cancer Med* 2019;8:155-64. doi:10.1002/cam4.1915

For peer review only - http://bmjopen.bmj.com/site/about/guidelines.xhtml

Declarations

Authors' contributions:

JB, GD, WL, RW, DC, IO, JK and JW led the conceptualisation of the work and the development of the design was led by JB, GD, WL, GA, BNGE, YT, MB, KL, RC and TW. With guidance from the other authors, BNGE wrote the first draft and all authors contributed to revising key content to develop the final version. All authors have seen and approve of the final manuscript.

For peer review only

Funding statement:

JB receives funding to support this work from NHMRC grants APP9100002 and APP1135048.

Competing interests statement

None to declare

For peer review only - http://bmjopen.bmj.com/site/about/guidelines.xhtml

List of tables and figures

Figure 1. Key elements of MDC

Figure 2. MDC interventions at transitions in cancer care

Figure 3. MDTM data collection template

Figure 4. Mapping of points of diagnosis and treatment decision-making in MDC

For peer review only

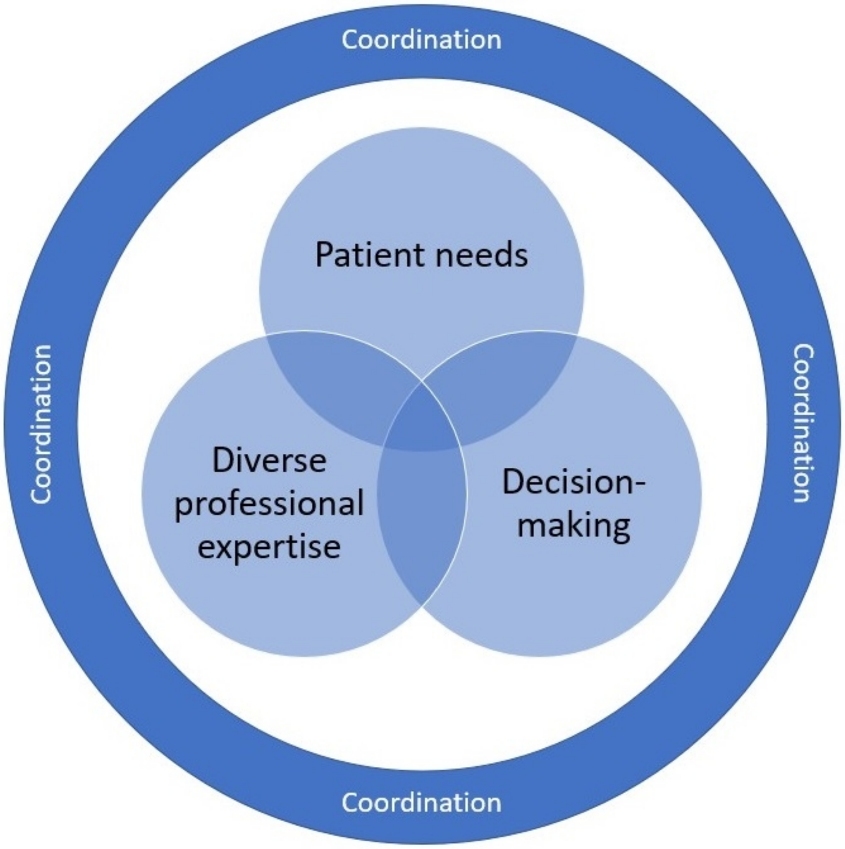

Source: Authors' conceptualisation of MDC

66x58mm (300 x 300 DPI)

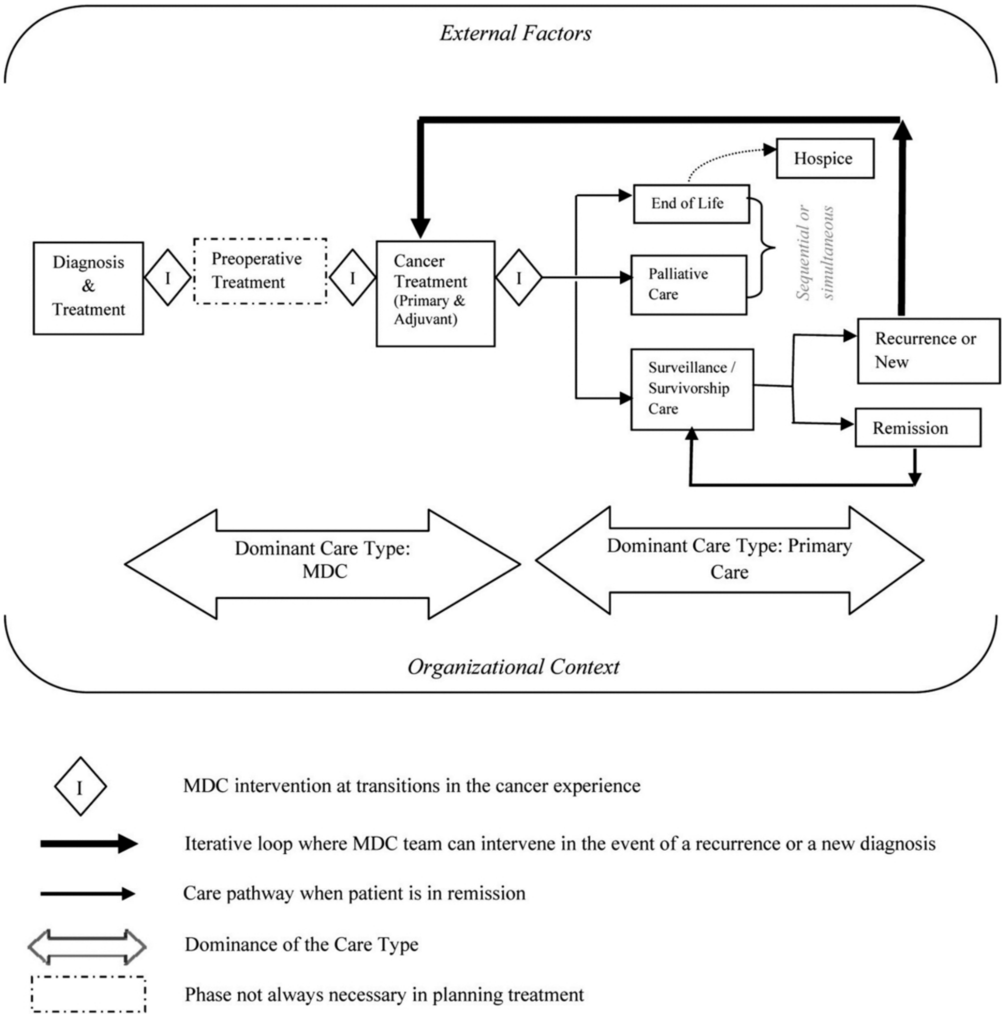

Source: Fennell et al. (2010)55 (adapted from Zapka et al., 2003)57

188x190mm (300 x 300 DPI)

MDTM & Hospital:<br>Date:	Notes
Attendance of health professional groups represented at MDT meetings by various health professional groups	
Chairperson and any meeting specific roles allocated	
Technological, equipment and administrative support in MDT meetings	
Organisation of the presentation of cases including<br>• The order<br>• Any prioritisation strategies<br>• Any patient inclusion criteria for meetings	
Clinical decision-making<br>• Discipline of professional input<br>• Types of information considered<br>• Evidence and/ or research incorporated into meetings	
Patient-centred care & Supportive care needs	
Organisation of any follow up tasks	

Source: Developed by authors based on Harris et al (2016)56 and Rankin et al. (2017)27

108x60mm (300 x 300 DPI)

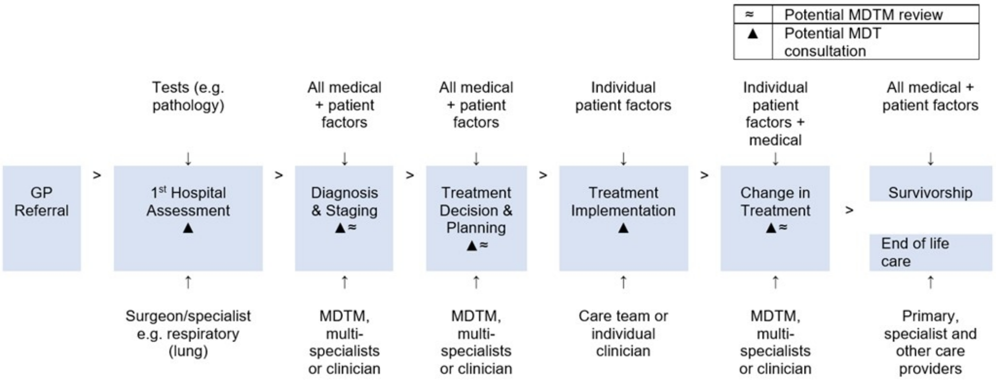

Source: Authors' adaption of Fennell et al. (2010)55

86x33mm (300 x 300 DPI)

COREQ (COnsolidated criteria for REporting Qualitative research) Checklist

A checklist of items that should be included in reports of qualitative research. You must report the page number in your manuscript where you consider each of the items listed in this checklist. If you have not included this information, either revise your manuscript accordingly before submitting or note N/A.

Topic	Item No.	Guide Questions/Description	Reported on Page No.
**Domain 1: Research team and reflexivity**			
Personal characteristics			
Interviewer/facilitator	1	Which author/s conducted the interview or focus group?	
Credentials	2	What were the researcher's credentials? E.g. PhD, MD	
Occupation	3	What was their occupation at the time of the study?	
Gender	4	Was the researcher male or female?	
Experience and training	5	What experience or training did the researcher have?	
Relationship with participants			
Relationship established	6	Was a relationship established prior to study commencement?	
Participant knowledge of the interviewer	7	What did the participants know about the researcher? e.g. personal goals, reasons for doing the research	
Interviewer characteristics	8	What characteristics were reported about the inter viewer/facilitator? e.g. Bias, assumptions, reasons and interests in the research topic	
**Domain 2: Study design**			
Theoretical framework			
Methodological orientation and Theory	9	What methodological orientation was stated to underpin the study? e.g. grounded theory, discourse analysis, ethnography, phenomenology, content analysis	
Participant selection			
Sampling	10	How were participants selected? e.g. purposive, convenience, consecutive, snowball	
Method of approach	11	How were participants approached? e.g. face-to-face, telephone, mail, email	
Sample size	12	How many participants were in the study?	
Non-participation	13	How many people refused to participate or dropped out? Reasons?	
Setting			
Setting of data collection	14	Where was the data collected? e.g. home, clinic, workplace	
Presence of non-participants	15	Was anyone else present besides the participants and researchers?	
Description of sample	16	What are the important characteristics of the sample? e.g. demographic data, date	
Data collection			
Interview guide	17	Were questions, prompts, guides provided by the authors? Was it pilot tested?	
Repeat interviews	18	Were repeat inter views carried out? If yes, how many?	
Audio/visual recording	19	Did the research use audio or visual recording to collect the data?	
Field notes	20	Were field notes made during and/or after the inter view or focus group?	
Duration	21	What was the duration of the inter views or focus group?	
Data saturation	22	Was data saturation discussed?	
Transcripts returned	23	Were transcripts returned to participants for comment and/or	

For peer review only

Topic	Item No.	Guide Questions/Description	Reported on Page No.
		correction?	
**Domain 3: analysis and findings**			
Data analysis			
Number of data coders	24	How many data coders coded the data?	
Description of the coding tree	25	Did authors provide a description of the coding tree?	
Derivation of themes	26	Were themes identified in advance or derived from the data?	
Software	27	What software, if applicable, was used to manage the data?	
Participant checking	28	Did participants provide feedback on the findings?	
Reporting			
Quotations presented	29	Were participant quotations presented to illustrate the themes/findings? Was each quotation identified? e.g. participant number	
Data and findings consistent	30	Was there consistency between the data presented and the findings?	
Clarity of major themes	31	Were major themes clearly presented in the findings?	
Clarity of minor themes	32	Is there a description of diverse cases or discussion of minor themes?	

Developed from: Tong A, Sainsbury P, Craig J. Consolidated criteria for reporting qualitative research (COREQ): a 32-item checklist for interviews and focus groups. *International Journal for Quality in Health Care*. 2007. Volume 19, Number 6: pp. 349 – 357

Once you have completed this checklist, please save a copy and upload it as part of your submission. DO NOT include this checklist as part of the main manuscript document. It must be uploaded as a separate file.

For peer review only
